# Differences in Flower Transcriptome between Grapevine Clones Are Related to Their Cluster Compactness, Fruitfulness, and Berry Size

**DOI:** 10.3389/fpls.2017.00632

**Published:** 2017-04-27

**Authors:** Jérôme Grimplet, Javier Tello, Natalia Laguna, Javier Ibáñez

**Affiliations:** Departamento de Viticultura, Instituto de Ciencias de la Vid y del Vino (Consejo Superior de Investigaciones Científicas, Universidad de La Rioja, Gobierno de La Rioja)Logroño, Spain

**Keywords:** *Vitis vinifera*, cluster architecture, phenotyping, transcriptomics, somatic variation

## Abstract

Grapevine cluster compactness has a clear impact on fruit quality and health status, as clusters with greater compactness are more susceptible to pests and diseases and ripen more asynchronously. Different parameters related to inflorescence and cluster architecture (length, width, branching, etc.), fruitfulness (number of berries, number of seeds) and berry size (length, width) contribute to the final level of compactness. From a collection of 501 clones of cultivar Garnacha Tinta, two compact and two loose clones with stable differences for cluster compactness-related traits were selected and phenotyped. Key organs and developmental stages were selected for sampling and transcriptomic analyses. Comparison of global gene expression patterns in flowers at the end of bloom allowed identification of potential gene networks with a role in determining the final berry number, berry size and ultimately cluster compactness. A large portion of the differentially expressed genes were found in networks related to cell division (carbohydrates uptake, cell wall metabolism, cell cycle, nucleic acids metabolism, cell division, DNA repair). Their greater expression level in flowers of compact clones indicated that the number of berries and the berry size at ripening appear related to the rate of cell replication in flowers during the early growth stages after pollination. In addition, fluctuations in auxin and gibberellin signaling and transport related gene expression support that they play a central role in fruit set and impact berry number and size. Other hormones, such as ethylene and jasmonate may differentially regulate indirect effects, such as defense mechanisms activation or polyphenols production. This is the first transcriptomic based analysis focused on the discovery of the underlying gene networks involved in grapevine traits of grapevine cluster compactness, berry number and berry size.

## Introduction

Grapevine (*Vitis vinifera* L.) is one of the most valuable horticultural crops in the world, with a total grape production of 77 million ton (2013, http://faostat3.fao.org). The value of any table grape, grape juice, or wine product relies fundamentally on disease-free and high quality fruits. Cluster compactness, an issue specific to grapevine, directly impacts fruit quality and disease susceptibility: Berries in compact clusters tend to ripe more asynchronously, impacting quality at harvest and compact cluster are also more susceptible to diseases, such as *Botrytis cinerea* (Molitor et al., 2012b).

Cluster compactness is a complex trait, resulting from the interaction of parameters related to cluster architecture and berry morphology, each contributing differently within a cultivar. Shavrukov et al. (2004) indicated the internode length of inflorescence rachis is the major trait responsible for inflorescence openness in four grape cultivars. However, a smaller berry size is responsible for loose cluster in Albariño (Alonso-Villaverde et al., 2008), while in other study, cluster density is correlated with the number of seeds per berry in the progeny of two wine grape cultivars (Bayo-Canha et al., 2012). More recently, our group has dissected the cluster compactness trait on a large set of table and wine cultivars (Tello et al., 2015). This exhaustive survey indicates that the berry number and the length of the rachis main axes (cluster architecture) are the most critical parameters for cluster compactness, followed by berry size. Each of these cluster compactness features is specific to different development stages. (i) Architecture related parameters are defined early. At the end of the first season summer, the primary latent bud contains a compressed shoot with inflorescence meristems, tendril and leaf primordia. In the second season, during initial stages of bud swelling, the inflorescence branch meristems can additionally ramify to form further inflorescence branch meristems that divide into a group of flower meristems (normally three). At that point, the inflorescence/cluster architecture is essentially set, as rachis elongation is limited after flowering (Coombe, 1995; Shavrukov et al., 2004). (ii) Final berry number in the cluster depends on the initial number of flowers and the fruit set rate that occurs after anthesis, although a compensation effect does exist (May, 2004). The initial number of flowers in the inflorescence is determined early in the second season, before bud burst, and it is noted that high temperatures at this stage decrease the number of flowers eventually formed (Ezzili, 1993). The availability of carbohydrate reserves in the trunk and roots (from the previous season) may also be a limiting factor (Bennett et al., 2002). Fruit set rate depends on the success of the pollination and fertilization processes, and also on the competition with other sink organs, mainly growing shoots. (iii) Two main factors are responsible for the size of the ripe berry at harvest: the cell number and their volume. Cell division is particularly active before anthesis and stops when the berry reach the lag phase, at the beginning of ripening (véraison). From that point only growth by cell enlargement occurs (Harris et al., 1968; Dokoozlian, 2000).

Little is known about the molecular basis or genetic factors responsible for differences in cluster compactness among grapevine cultivars and clones. Experimental treatments to reduce cluster compactness involve enlarging inflorescence main axes, reducing fruit set, and/or reducing berry size. Plant hormones control grapevine reproductive development and flowering timing through the gibberellin:cytokinin balance. Gibberellins mediate the formation of the inflorescence axis, while cytokinins regulate the differentiation into flowers and are specifically needed for the growth of pistil (Pool, 1975). ABA concentration is high before anthesis, and auxin transport is needed to avoid abscission and promote fruit set (Kühn et al., 2014). The application of the gibberellins inhibitor prohexadione-Ca causes a loosening effect by reducing berry size and/or number of berries, likely through disturbing pollination and cell division processes (Molitor et al., 2011; Schildberger et al., 2011). The application of gibberellic acid pre-bloom promotes the growth of the inflorescence (Hed et al., 2011; Molitor et al., 2012a), while gibberellin treatments during bloom reduce fruit set and increase berry size (Ben-Tal, 1990).

The availability of the grapevine genome sequence (Jaillon et al., 2007; Velasco et al., 2007) allowed high throughput studies of the grapevine that are leading to an increased knowledge of the molecular events occurring behind physiological processes. In this work we performed transcriptomic analyses of Garnacha Tinta clones, with stable differences in specific compactness-related parameters (berry number, berry size), to identify genes and gene networks involved in cluster compactness characteristics. From this transcriptomics study, 183 candidate genes were selected for an association analysis in a collection of grapevine varieties (Tello et al., 2016).

## Methods

### Plant material

In the early 2000s, Gobierno de La Rioja prospected the entire Rioja region and collected hundreds of grapevine (*Vitis vinifera* L.) plants of different cultivars, usually old plants and/or plants with particular characteristics. Each of these plants was multiplied by cuttings and grafted on Richter 110 rootstock. Five clonal grafted vines per original plant were planted together in a single plot at the experimental vineyard of La Grajera (Logroño, La Rioja). This clone collection includes 501 clones from Garnacha Tinta, which were screened for cluster compactness, in sequential steps. First, the compactness of all the clones was visually assessed. Then, nine clones were selected for phenotyping during the next season and six of these were also phenotyped during a second and third season. Finally, four of these clones, two with compact clusters (“compact clones”) and two with loose clusters (“loose clones”) were selected for transcriptomic analysis.

### Phenotyping

In three successive seasons, six selected Garnacha Tinta clones were phenotyped for several variables related to cluster compactness using five clusters per clone as described by Tello and Ibáñez (2014). All the clones were subjected to pair-wise comparisons for phenotypic variables grouped in four categories: plant (e.g., fertility), cluster architecture (e.g., cluster length), fruitfulness (berry number and seed number) and berry size (Supplementary Table 1). Clone pairs differing only in one category were favored, but the most selective criterion was consistency over the seasons for the observed significant pair-wise differences, and some clone comparisons with non-consistent differences were discarded. Finally, four clones (368, 906, 1134, and 1154) were used for transcriptome analysis (Supplementary Table 2).

### Experimental design and sampling for transcriptome analysis

**Figure 1:**
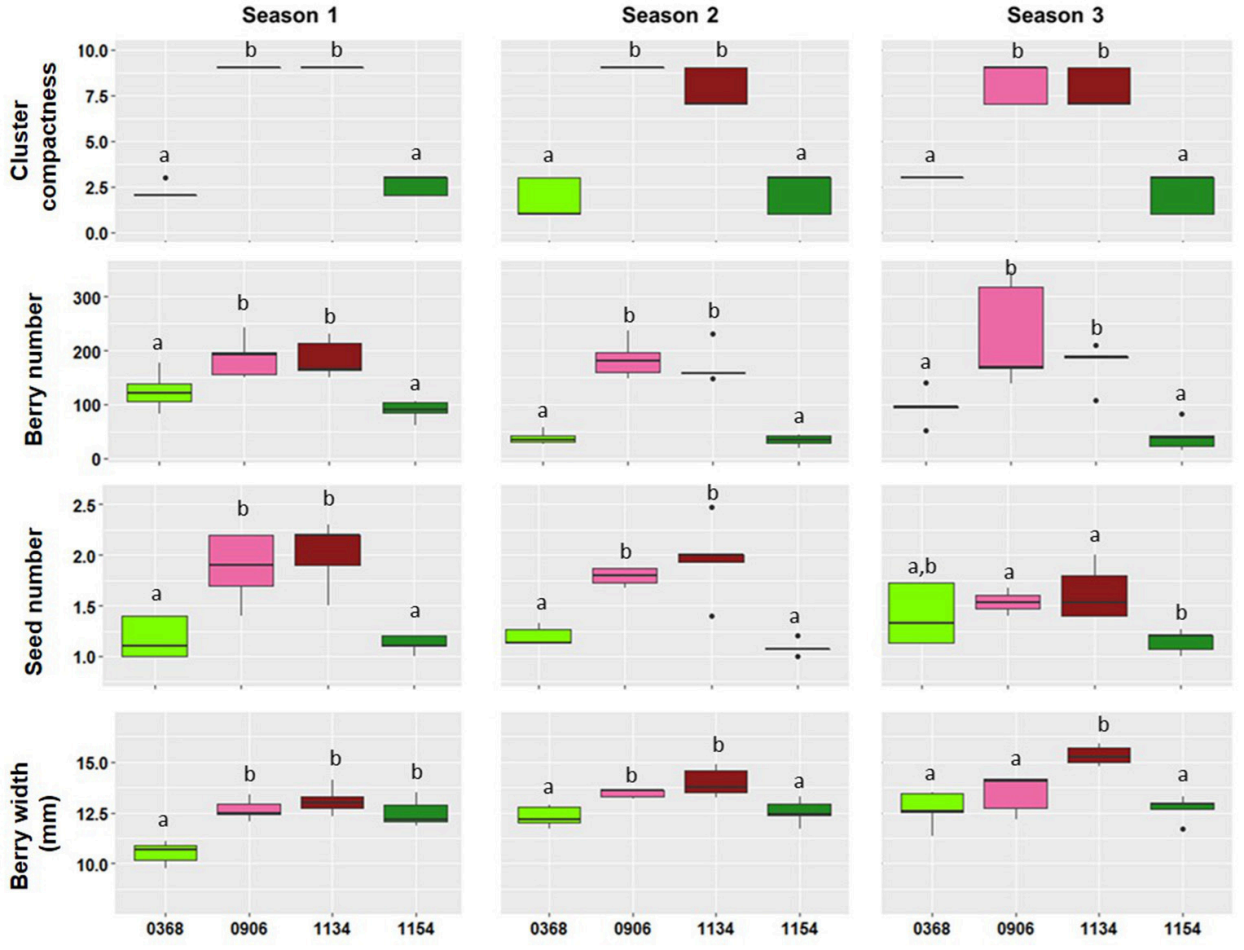
**Boxplots showing the phenotypic distribution of four traits [Cluster compactness (OIV-rating), Berry number per cluster, Seed number per berry, and Berry width (mm)] for the four clones analyzed (368, 906, 1134, and 1154) during three different seasons**. Different lowercase letters within a plot indicate a significant difference among clones according to Fisher's LSD-tests (*p* ≤ 0.05).

The experimental design was determined in accordance with the significant pair-wise differences consistently observed between the selected clones of Garnacha Tinta over the three seasons (Supplementary Table 1, Figure 1). Organs and stages were sampled based on specific differential parameters: berry number, seed number and berry size (Table 1). For berry number, flowers were sampled at the end of flowering (E-L 26, Coombe, 1995) before possible abscission or set (Table 1; comparisons G1-26, G2-26, G3-26, and G4-26). Seed number is also determined at that step since it depends on the success of pollination. Spring buds at budburst were sampled in two clones to study the initial number of flowers (E-L 3: comparison G4-03), when flowers start to differentiate (Pouget, 1981; Dunn and Martin, 2000).

**Table 1:** **Experimental design for each of the comparisons performed between Garnacha Tinta clones**.

**Comparison**	**Clone 1**	**Clone 2**	**Significant phenotypic differences (*P* < 0.05) three seasons**	**Organ sampled**	**Sampling stage (modified E-L)**	**Pair-wise comparison code**
G1	1134 (C)	368 (L)	N° berries, Berry size	Flowers	End of flowering (E-L 26)	G1-26
				Berries	Start of véraison (E-L 34)	G1-34
G2	1134 (C)	1154 (L)	N° berries, N° seeds	Flowers	End of flowering (E-L 26)	G2-26
G3	906 (C)	368 (L)	N° berries	Flowers	End of flowering (E-L 26)	G3-26
G4	906 (C)	1154 (L)	N° berries, N° seeds	Spring buds	Bud burst (E-L 03)	G4-03
				Flowers	End of flowering (E-L 26)	G4-26

*Organs and sampling stages for transcriptomics analyses were chosen based on the stable phenotypic differences found in three seasons. C, Compact clone; L, Loose clone*.

Berry size is determined by cell division and cell expansion. So, analyses for berry size were carried out on flowers at the end of flowering, when cell division is active (E-L 26; comparison G1-26), and on green berries at the beginning of véraison (E-L 34), when cell division is complete and berry enlargement by cell expansion begins (Dokoozlian, 2000; comparison G1-34). As berries are in different developmental stages within the same cluster at a given time, sampled berries were classified according to their density by flotation on NaCl solutions (Carbonell-Bejerano et al., 2016). Green berries floating in a solution of 80 g/l NaCl and sinking in a solution of 60 g/l NaCl were selected.

Three replicate samples were collected from different vines. After collecting, samples were immediately frozen in liquid nitrogen, and then kept in the laboratory at −80°C until RNA extraction.

### RNA extraction and microarray hybridization

Total RNA was extracted from samples using the Spectrum plant total RNA kit (Sigma, www.sigmaaldrich.com) as recommended by manufacturer. DNase I digestion was carried out with the RNase-Free DNase Set (QIAGEN). RNA integrity and quantity were assessed with a Nanodrop 2000 spectrophotometer (Thermo Scientific) and an Agilent's Bioanalyzer 2100. Microarray hybridizations were performed at the Genomics Unit of the National Centre for Biotechnology (CNB-CSIC, Madrid).

Synthesis of cDNA, labeling, hybridization, and washing steps were performed according to the NimbleGen arrays user's guide. Each sample was hybridized to a NimbleGen microarray 090818 Vitis exp HX12 (Roche, NimbleGen), which contains probes targeted to 29,549 predicted grapevine genes and 19,091 random probes as negative controls. Images were analyzed using NimbleScan v2.6 software (Roche), which produces.xys files containing the raw signal intensity data for each.

### Microarray data processing

The data discussed in this publication have been deposited in NCBI's Gene Expression Omnibus (Edgar et al., 2002) and are accessible through GEO Series accession number GSE67708 (www.ncbi.nlm.nih.gov/geo/query/acc.cgi?acc=GSE67708).

Raw intensity values were processed using the R package oligo (Carvalho and Irizarry, 2010). Individual probes raw expression values were computed from.xys files and the in house pd info builder package pd.vitus.exp.vitnames designed to fit the 12Xv1 annotation nomenclature. Normalization was performed with Robust Multi-Array Average (RMA; Irizarry et al., 2003). Resulting RMA expression values were log_2_-transformed. Distributions of expression values processed via RMA of all arrays were very similar with no apparent outlying arrays.

### Microarray data analysis

Each condition (clone × stage/organ) was performed in three biological replicates. Differential expression analyses of the comparisons presented in Table 1 were performed with the ebayes (Smyth, 2004) method from the package limma in R. The cutoff of differentially expressed genes was set to a *p* < 0.05 after Benjamini-Hochberg correction with at least a 2-fold ratio difference of expression. Principal component analysis (PCA) was performed in R using the pca package with the ppca method. Hierarchical clustering was performed using MultiExperiment Viewer (Saeed et al., 2003) based on Pearson's correlation and using the average linkage option and optimal gene ordering. The stringent set was obtained by clustering genes with a distance threshold < 0.05. The tolerant set was obtained by clustering genes with a distance threshold < 1.5.

To identify the biological functions over-represented within selected probe sets, functional enrichment analyses were performed using FatiGO (Medina et al., 2010; *P* < 0.05). Functional categories were based on manual annotation of 12Xv1 grape genome assembly, described in Grimplet et al. (2012).

### Cytoscape/vitisnet analysis

Expression data were uploaded in Cytoscape version 3 (Shannon et al., 2003) and analyzed with VitisNet (Grimplet et al., 2012). According to FatigoGO analysis, networks related to enriched categories were selected for manual inspection. The visual style in the figures was designed to best represent changes in flower by including notifications of the genes over-expressed in compact or loose clones. A color gradient was used depending on the presence of the differentially expressed gene (DEG) in 2 or 3 (light color) to 4 (dark color) comparisons, to have a visual representation of the DEG degree of recurrence in the comparisons. Network ID corresponds to the VitisNet ID (Grimplet et al., 2009).

## Results and discussion

### Phenotyping and comparison of the clones

In a multi-cultivar framework, our group identified the major morphological factors influencing the cluster compactness trait (Tello and Ibáñez, 2014; Tello et al., 2015). Different variables, classified within four major groups (plant, cluster architecture, fruitfulness (berry and seed number) and berry size), were phenotyped in a large set of diverse cultivars, and it was concluded that the length of the cluster main axes and berry number were the main discriminant variables for cluster compactness, followed by the berry size. In the present work, a similar set of variables was used to study Garnacha Tinta clones and only clone pairs consistently differing in selected variables were used for analyses. Cluster compactness remained consistent through the seasons, but some of the significant differences observed the first season were not stable over the three seasons. Therefore, some clone comparisons were discarded. Finally, four clones of Garnacha Tinta, two loose (368 and 1154) and two compact clones (906 and 1134) were used for transcriptome analysis (Table 1, Supplementary Table 2).

Similar to cluster compactness, the berry number showed very consistent differences through the seasons in the four clone comparisons (Table 1, Figure 1, Supplementary Table 1). The compact clones produced significantly tighter clusters than loose clones and carried a significantly greater number of berries in all the comparisons studied during the three seasons.

Berry number was the only differential variable in comparison G3, but in the remaining comparisons additional seasonally stable differences appeared in other traits. Thus, comparison G1 was selected to examine the transcriptional changes observed between a loose clone and a compact clone with significant differences in berry number and berry size. The compact clone (1134) produced more and larger berries than the loose clone (368) (Figure 1, Supplementary Table 1).

Finally, comparisons G2 and G4 were selected to examine differences in global gene expression related to the two variables included in the fruitfulness category: berry number and seed number. In these comparisons, the compact clones always had more berries per cluster and more seeds per berry than the loose clones (Figure 1, Supplementary Table 1). This was expected, as both the number of seeds and fruit set are related to pollination and fertilization, and flower fate (abscission or berry set) greatly depends on the existence of at least one fertilized ovule in the flower (Kassemeyer and Staudt, 1982).

### Global gene expression data

**Figure 2:**
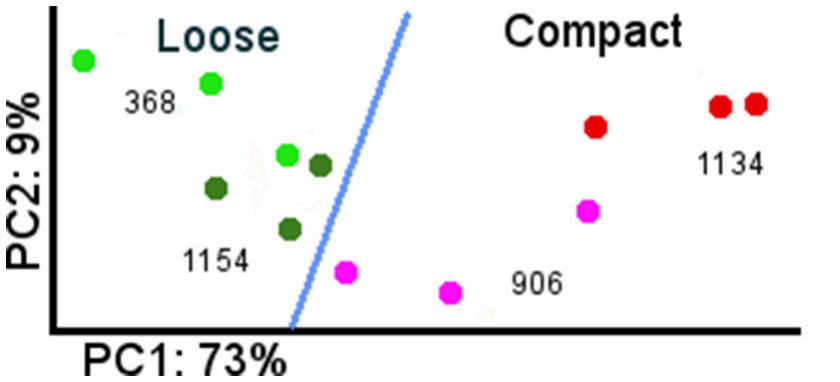
**PCA of global gene expression data at flowering between loose and compact clones**. Green dot: Garnacha Tinta loose clone 368. Dark green dot: Garnacha Tinta loose clone 1154. Pink dot: Garnacha Tinta compact clone 906. Red dot: Garnacha Tinta compact clone 1134.

The greatest number of differentially expressed genes (DEG) between clones was in flower at the end of flowering (E-L 26), while very few differences could be seen in spring buds (G4-03) (Table 2). Figure 2 represents the first two axes of a PCA of the expression data obtained for the four studied clones at the end of bloom, E-L 26. The first component of the PCA represented 73% of the total variation and seemed related to compactness. Component 2 accounted for 9% of the total variability, separating genotypes.

**Table 2:** **Number of differentially expressed genes (DEG) at every time point for every comparison Compact vs. Loose clone**.

**Over-expressed in:**	**G1-26**	**G1-34**	**G2-26**	**G3-26**	**G4-03**	**G4-26**	**Compact vs. loose at E-L 26**
Compact	2600	204	2066	1565	5	515	400
Loose	2720	296	2380	848	10	150	70
Total	5320	500	4446	2413	15	665	470

The replicates from compact and loose clones were clearly separated, however clones of the same compactness presented a large variation. There were differences in the sampling dates to match physiological state but they did not seem to be related to the variation, since more variability could be observed between some replicates sampled the same day (data not shown). It is known that, within the same inflorescence, there are flowers in different stages of development, including those with already fertilized ovules, others with fertilization in progress, and others that have not been fertilized and probably will drop (Kühn et al., 2014). These flower stages are not visually distinguishable during sampling, but their transcriptomic profiles are probably different, because there are evidences in grapevine indicating that pollination rapidly modifies gene expression (Kühn and Arce-Johnson, 2012). The proportion of flowers in each of those stages would vary differently between clones, partially explaining the consistent differences in the number of berries observed in the four comparisons. This is probably the major cause for the gene expression differences observed in the four pair-wise comparisons at the end of flowering (Table 2, E-L 26).

At that stage, a greater number of DEG was observed in comparisons involving the compact clone 1134 (G1-26 and G2-26, 5320 and 4446 genes, Table 2) than in comparisons with the other compact clone, 906 (2413 and 665 genes). That variation may be the result of an asynchronous floral development in clone 1134, which would lead to the sampling of slightly different flower stages in the compared clones. This would be supported by the high number of DEG (1607 genes) observed at E-L 26 between clones 1134 and 906 (Table 2). This difference is reduced later, as illustrated in comparison G1-34, where the two clones involved (1134 and 368) reached similar transcriptomes, with a minimal number of differentially expressed genes between them at E-L 34.

In comparison G1, in addition to a different number of berries, a consistent difference in berry size was observed, unlike in G2. So, the differentially expressed genes found in G1-34 and (partly) in G1-26 could be related to the fact that clone 368 showed a smaller berry size than 1134 during the three studied years.

The number of significant DEG obtained for the stage end of flowering (E-L 26) was much lower in loose clones (368 + 1154) than in compact clones (1134 + 906) when all comparisons are considered. Only 70 gene transcripts were more abundant in the loose clones and 400 in the compact clones (Table 2). Many genes, however, were differentially expressed between the compact clone 1134 and any loose clone (2051 genes showed a greater expression in loose clones, 1683 in the compact clone).

### Functional categories analysis

Functional categories enrichment analysis was performed in order to identify the main mechanisms impacted in cluster compactness and their related traits. Since PCA showed greatest differences in expression pattern in clones 1134 and 368, analyses were performed considering several situations: group 1 includes the genes differentially expressed in all comparisons at E-L 26 (Table 3) that are specifically related to compactness independent of the clone (400 over-expressed genes in compact clones, 70 in loose clones); group 2 contains the genes specifically regulated in the most extreme compact clone 1134, i.e., differentially expressed in G1 and G2: genes expressed in clone 1134 (2051 over-expressed genes) vs. all the loose (1703 over-expressed genes) (Table 4); and group 3 comprises the genes specifically regulated in the most extreme loose clone 368, i.e., differentially expressed in G1 and G3 comparisons: over-expressed genes in clone 368 (1400) vs. all the compact (560) (Table 5). If a category is enriched in both the compact and loose clones, this means that there are distinct genes from that category represented in a larger proportion in both sets than in the whole transcriptome. As indicated in 4.2, for group 1 few genes were differentially expressed in the loose clusters considering all comparisons at E-L 26; therefore, only two categories were enriched in the loose clustered type (Table 3). Overall, several patterns of expression emerged from the three enrichment analyses. Within the functional categories related to the metabolism, several functional categories indicate a dramatic shift of expression of genes involved in the metabolism. The category related to cell growth and death was over-represented in all the clones with compact clusters (Tables 3–5). Cytoskeleton, chromosome organization and biogenesis and DNA metabolism were also over-abundant in the compact clones (Tables 3–5), indicating a possible greater cellular replication activity in the compact clones. Categories related to cell wall showed clear specificity of transcript expression in either compact or loose clones. Pectin-related categories were only over-represented in the compact clones and cellulose biosynthesis in the loose clones (Tables 4, 5). Phenylpropanoids-related categories showed dramatic changes in gene expression, the phenylpropanoid metabolism category was over-represented in both the compact and loose clones (Tables 4, 5). The lignin biosynthesis category seems more abundant in the compact clone (1134) when compared with both loose clones (Table 4) and the loose clone 368 when compared with both compact clones (Table 5). Terpenoids and alkaloids categories also seemed to be over-represented in the loose clones (Tables 4, 5). In addition the plant-pathogen interaction category was also over-represented in the loose clones vs. 1134 (Table 4). Several categories related to hormone signaling were also over-represented in the loose clones, such as Auxin, brassinosteroids, cytokinins, jasmonate, and ethylene signaling (Tables 4, 5). Transporters showed a balanced pattern; however, oxygen transport was more abundant in the loose clones (Tables 4, 5). Ion transport-related categories were also over-represented in both types of clones.

**Table 3:** **Over-represented functional categories in all E-L 26 comparisons with ***P*** < 0.05**.

	**Compact**	**Loose**
**01 Cellular process**	1.23	
01.01 Cell growth and death	2.04	
01.01.04 Cell growth	3.08	
01.02 Cellular component organization and biogenesis	1.34	
01.02.01 Cell wall organization and biogenesis	1.65	
01.02.01.01 Cell wall metabolism	1.68	
01.02.01.01.02 Cell wall catabolism	2.86	
01.02.02 Nucleus	2.72	
01.02.02.01 Chromosome organization and biogenesis	2.72	
01.02.02.01.01 Chromatin assembly	3.88	
**04 Metabolism**		
04.01 Cellular metabolism		
04.01.08.03 Oxidation reduction. Copper oxidase family	4.57	
04.02 Primary metabolism		
04.02.01.06 Aromatic amino acid metabolism	1.46	
04.02.01.06.01 Aromatic amino acid biosynthesis	2.16	
04.02.08.01.01 Nucleic acid metabolism. DNA metabolism	1.80	
04.02.08.01.01.03.02.01 Base excision repair	3.57	
04.02.08.01.01.05 DNA replication	3.68	
04.03 Metabolism. Secondary metabolism		
04.03.02.01 Aromatic compound biosynthesis	3.24	
**05 Regulation overview**	0.87	
05.01 Regulation of cell cycle	2.40	
05.02 Regulation of gene expression	0.69	
05.02.02 Regulation of transcription	0.73	
05.02.02.01 Transcription factor	0.76	
05.02.02.01.44 MYB family transcription factor	1.91	
**07 Signaling**		
05.02.02.01.49 PLATZ family transcription factor	4.16	
07.01.02 Signaling. Hormone Signaling. Auxin Signaling	1.50	
07.01.02.04 Auxin-mediated Signaling pathway	1.73	
07.02.09 Protein kinase		1.77
**09 Unknown**	2.77	3.21

*Values are expressed as log2 ratio group/genome*.

**Table 4:** **Over-represented functional categories in G1 and G2 comparisons with ***P*** < 0.05**.

	**Compact**	**Loose**
**01 Cellular process**	2.2	
01.01 Cell growth and death	3.6	
01.01.04 Cell growth	4.1	
01.02 Cell. component org. and biogen.	2.5	
01.02.01 Cell wall org. and biogenesis	2.8	
01.02.01.01 Cell wall metabolism	2.7	
01.02.01.01.01 Cell wall biosynthesis		4.6
01.02.01.01.01.02 Cellulose biosynthesis		6.9
01.02.01.01.02 Cell wall catabolism	3.7	
01.02.01.01.03 Cell wall modification	2.9	
01.02.01.01.03.02 Pectin modification	3.0	
01.02.01.02 Cell wall structural protein	3.8	
01.02.02 Nucleus	3.3	
01.02.02.01 Chrom. org. and biogenesis	3.3	
01.02.02.01.01 Chromatin assembly	5.4	
01.02.03 Cytoskeleton org. and biogen.	3.6	
01.02.03.03 Microtub. org. and biogen.	5.1	
01.02.03.03.01 Microtubule-driven mov.	6.8	
**04 Metabolism**		
04.01 Cellular metabolism		
04.01.08.03 Copper oxidase family	8.6	
04.02 Primary metabolism		
04.02.02 Carbohydrate metabolism		
04.02.02.08 Polysaccharide metabolism		
04.02.02.08.01 Beta-1,3 glucan met.	3.4	
04.02.02.08.01.01 Beta-1,3 glucan cat.	4.2	
04.02.07.05 Steroid metabolism	2.6	
04.02.07.05.01 Steroid biosynthesis	3.1	
04.02.08.01 Nucleic acid metabolism		
04.02.08.01.01 DNA metabolism	2.0	
04.02.08.01.01.05 DNA replication	5.4	
04.03 Secondary metabolism	1.5	1.7
04.03.01 Prim. amino acids deriv. met.		
04.03.01.01 Alkaloid metabolism		
04.03.01.01.01 Alkaloid biosynthesis		3.9
….01 Monoterp. indole alkaloid bioS		5.7
04.03.04 Phenylpropanoid met.	1.9	2.2
04.03.04.01 Flavonoid metabolism		
04.03.04.01.01 Flavonoid biosynth.		
04.03.04.01.01.01 Anthoc. biosynth.	3.1	
01 Anthoc.-glycoside bioS.	3.4	
04.03.04.03 Lignin metabolism	4.5	
04.03.04.05.Stilbenoid metabolism		7.1
04.03.04.05.01 Stilbenoid biosynth.		7.1
04.04 Single reactions	3.4	
**05 Regulation overview**	1.3	
05.01 Regulation of cell cycle	4.8	
5.02 Regulation of gene expression		
05.02.02 Regulation of transcription		
05.02.02.01.03 AP2 family		5.7
05.02.02.01.03.02 ERF subfamily		13.2
05.02.02.01.44 MYB family		4.4
**06 Response to stimulus**		1.7
06.02 Stress response		1.7
06.02.01 Abiotic stress response		
06.02.01.07 Oxidative stress response	2.2	
06.02.02.Biotic stress response		1.8
06.02.02.03 Plant-pathogen interact.		2.1
06.02.02.03.01 R proteins		2.4
**07 Signaling**		2.1
07.01 Hormone Signaling		1.8
07.01.04.01 Cytokinin metabolism		6.3
07.01.05 Ethylene Signaling		3.9
07.01.05.03 Ethylen.-med. Sign. path.		4.1
07.02 Signaling pathway		2.2
07.02.09 Protein kinase		3.0
07.02.12 Signaling receptor	9.0	
**08 Transport overview**		
08.02.01.49 Chloride Carrier/Channel		17.8
08.09 Incomp. charact. transport sys.	2.1	
08.09.01 transp. of unk. bioch. mech.	2.6	
08.09.01.10 Iron/Lead Transporter	4.7	
08.09.01.10.01 Oxi.-dep Fe2+ Transp.	4.7	
08.12.01 Oxygen transport		18.0
08.13.01.01 Chloride transport		14.2
**09 Unknown**	6.4	4.9

*Values are expressed as log2 ratio group/genome*.

**Table 5:** **Over-represented functional categories in G1 and G3 comparisons with ***P*** < 0.05**.

	**Compact**	**Loose**
**01 Cellular process**	2.2	
01.01 Cell growth and death	3.9	
01.02 Cellular component org. and biog.	2.3	
01.02.01 Cell wall org. and biogenesis	2.1	
01.02.01.01 Cell wall metabolism	2.2	1.7
01.02.01.01.01.02 Cellulose biosynthesis		3.2
01.02.01.01.02 Cell wall catabolism	3.6	
01.02.01.01.02.03 Pectin catabolism	5.9	
01.02.01.01.03 Cell wall modification	2.2	
01.02.02 Nucleus	3.3	
01.02.02.01 Chrom. org. and biogen.	3.3	
01.02.02.01.01 Chromatin assembly	5.0	
01.02.03 Cytoskeleton org. and biogen.	3.7	
01.02.03.02 Actin org. and biogenesis	2.8	
01.02.03.03 Microtubule org. and biogen.	5.0	
01.02.03.03.01 Microtubule-driven mov.	6.8	
**04 Metabolism**		
04.01 Cellular metabolism		2.0
04.01.01 Amino acid derivative met.		
04.01.01.01 Cyanoamino acid metabolism		4.1
04.01.06 Nitrogen and sulfur metabolism		1.9
04.01.06.01 Nitrogen metabolism		2.0
04.01.08 Oxidation reduction		1.8
04.01.08.04 Cytochrome P450 oxidored.		1.9
04.01.10.01 Phytoalexin biosynthesis		8.9
04.02.02.06.01 Amino sugar metabolism		2.7
04.02.02.08.01.01 Beta-1,3 glucan cat.	3.4	
04.02.02.08.02.01.04 Starch cat. inhibitor		10.0
04.02.04 Coenz. and prosthetic gr. met.		
04.02.04.04 Pept. deriv. compounds bioS.		2.1
04.02.04.04.01 Glutathione metabolism		2.1
04.02.05.02 Tetrapyrrole metabolism		3.1
04.02.06.06 Storage proteins		3.6
04.02.07.06.01 Wax biosynthesis		4.0
04.02.08.01.01 DNA metabolism	2.7	
…03 DNA recomb. and repair	2.4	
04.02.08.01.01.03.02 repair	2.3	
01 Base excision repair	5.9	
04.02.08.01.01.05 DNA replication	6.0	
04.02.10.01.01.02 HSP-med. prot. folding	2.1	
04.02.10.03.03 Protease inhibition		7.0
04.03 Secondary metabolism		1.9
04.03.01.01.01 Alkaloid biosynthesis		2.3
….03.01 Monoterp. indole alkaloid bioS.		3.5
04.03.04 Phenylpropanoid metabolism		2.9
04.03.04.01.01.01 Anthocyanin bioS.	2.8	
04.03.04.03 Lignin metabolism		5.9
04.03.04.04 Phenylpropanoid bioS.		3.7
04.03.04.05 Stilbenoid metabolism		13.0
04.03.04.05.01 Stilbenoid biosynthesis		13.0
04.04 Single reactions		4.3
**05 Regulation overview**	1.4	
05.01 Regulation of cell cycle	5.1	
05.02.02.01.03 AP2 family		2.9
05.02.02.01.03.02 ERF subfamily		3.3
05.02.02.01.11 bHLH fam. transc. factor	2.4	
05.02.02.01.44 MYB fam. transc. factor	2.5	2.1
05.02.02.01.66 WRKY fam. transc. fact.		4.2
**06 Response to stimulus**		1.7
06.02.01 Abiotic stress response		2.0
06.02.01.07 Oxidative stress response		2.7
06.02.02 Biotic stress response		1.4
**07 Signaling**		1.9
07.01 Hormone Signaling		1.6
07.01.02.01 Auxin metabolism		2.4
07.01.04 Cytokinin Signaling		2.3
07.01.04.01 Cytokinin metabolism		3.2
07.01.05 Ethylene Signaling		2.6
07.01.07 Jasmonate salicylate signaling		2.2
07.01.07.01 Jasmonate Signaling		2.3
07.02 Signaling pathway		2.0
07.02.09 Protein kinase		2.9
**08 Transport overview**		
08.02.01.07.14 Plant Org. Permease		6.0
08.02.01.49 Chloride Carrier/Channel		8.5
08.09 Incomp. char. transport systems		2.2
08.09.01.10.01 Oxid.-dep Fe2+ Transp.	2.5	5.6
08.12.01 Oxygen transport		11.3
08.13.01 Anion transport		2.3
08.13.01.01 Chloride transport		6.8
08.14.08 Nucleotide transport		4.9
**09 Unknown**	6.2	4.0

*Values are expressed as log2 ratio group/ genome*.

### Vitisnet analysis indicates metabolic pathways related to cluster compactness

**Figure 3:**
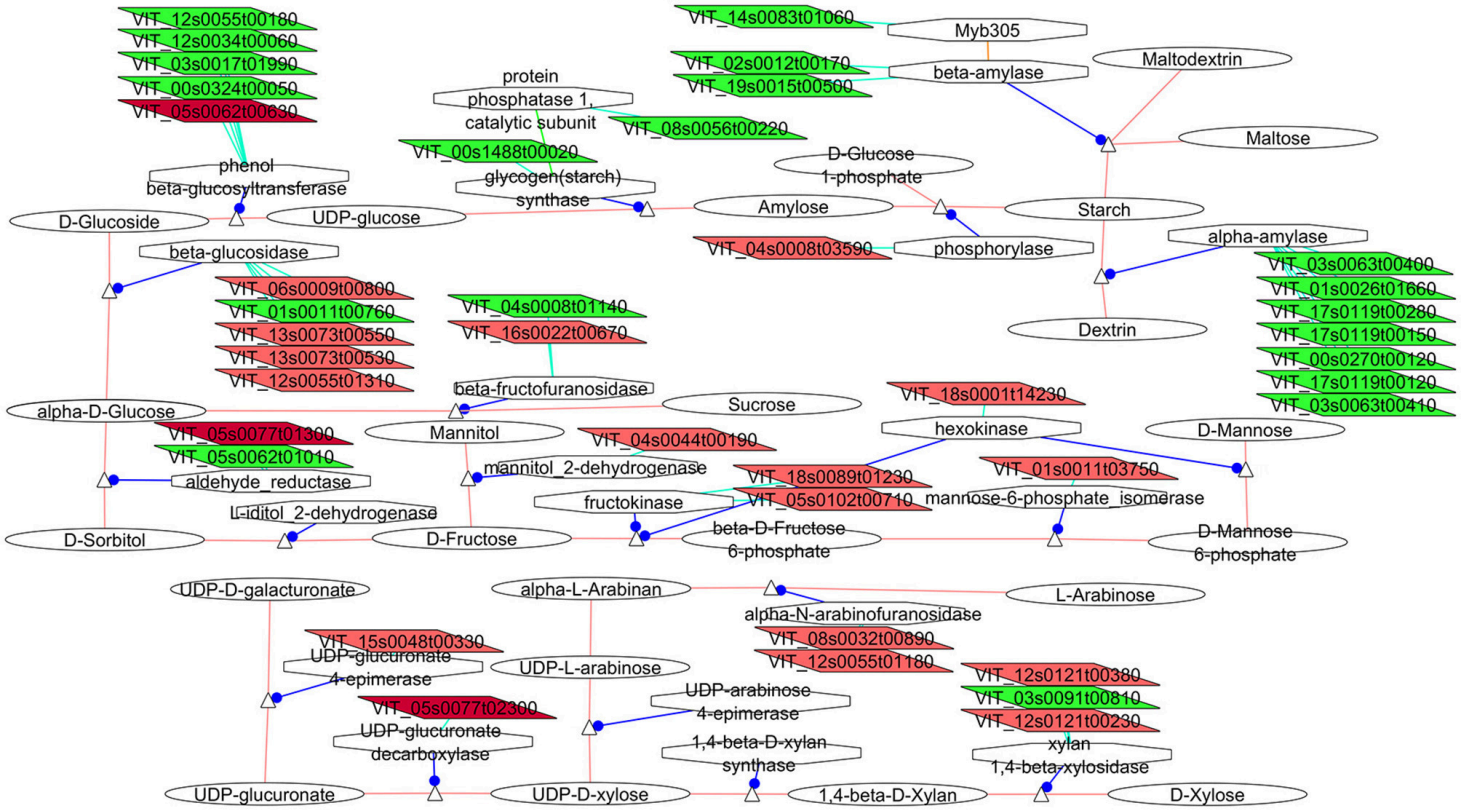
**Adapted Cytoscape networks including transcripts differentially expressed in flowers between loose and compact clones related to carbohydrates metabolism**. Genes over-expressed in compact clones in all comparisons are in dark red. Genes over-expressed in compact clones in 2 or 3 comparisons are in red. Genes over-expressed in loose clones in all comparisons are in dark green. Genes over-expressed in loose clones in 2 or 3 comparisons are in green. Figure is adapted from networks 10500, 10051, and 1052 from Grimplet et al. (2009).

**Figure 4:**
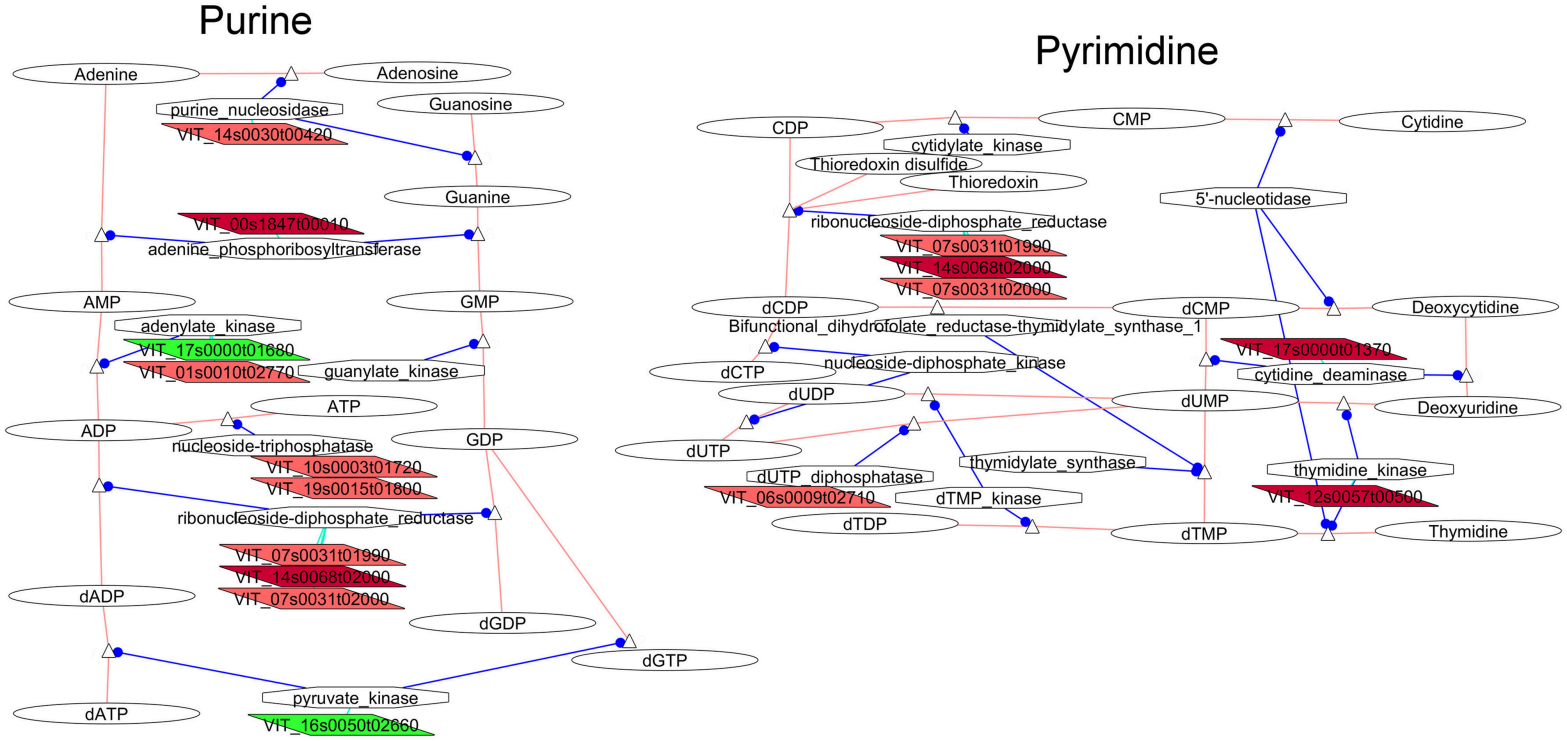
**Adapted Cytoscape networks including transcripts differentially expressed in flowers between loose and compact clones related to nucleic acid metabolism**. Genes over-expressed in compact clones in all comparisons are in dark red. Genes over-expressed in compact clones in 2 or 3 comparisons are in red. Genes over-expressed in loose clones in all comparisons are in dark green. Genes over-expressed in loose clones in 2 or 3 comparisons are in green. Figure is adapted from networks 10230 and 10240 from Grimplet et al. (2009).

**Figure 5:**
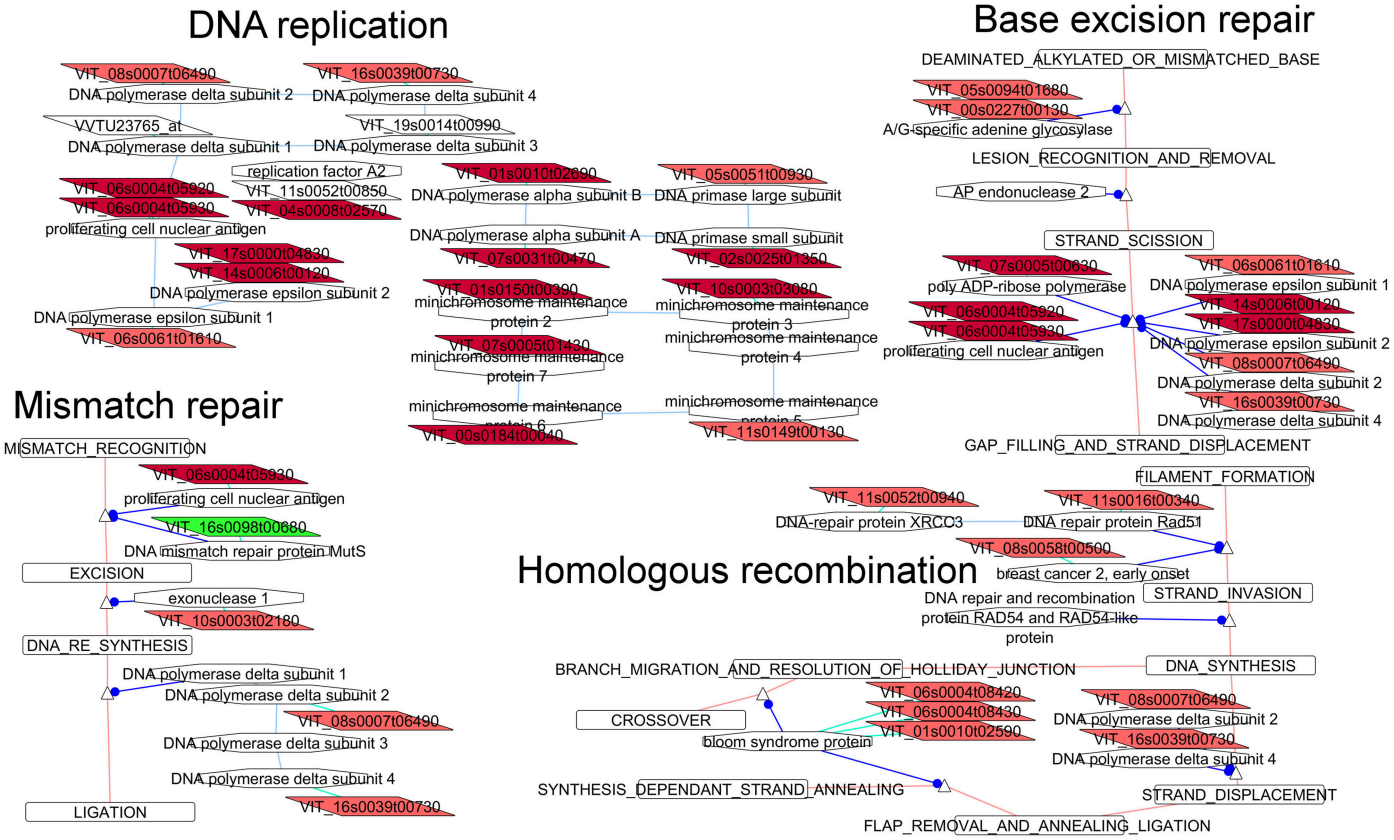
**Adapted Cytoscape networks including transcripts differentially expressed in flowers between loose and compact clones related to DNA repair metabolisms**. Genes over-expressed in compact clones in all comparisons are in dark red. Genes over-expressed in compact clones in 2 or 3 comparisons are in red. Genes over-expressed in loose clones in all comparisons are in dark green. Genes over-expressed in loose clones in 2 or 3 comparisons are in green. Figure is adapted from networks 23030, 23410, 23420, and 23430 from Grimplet et al. (2009).

**Figure 6:**
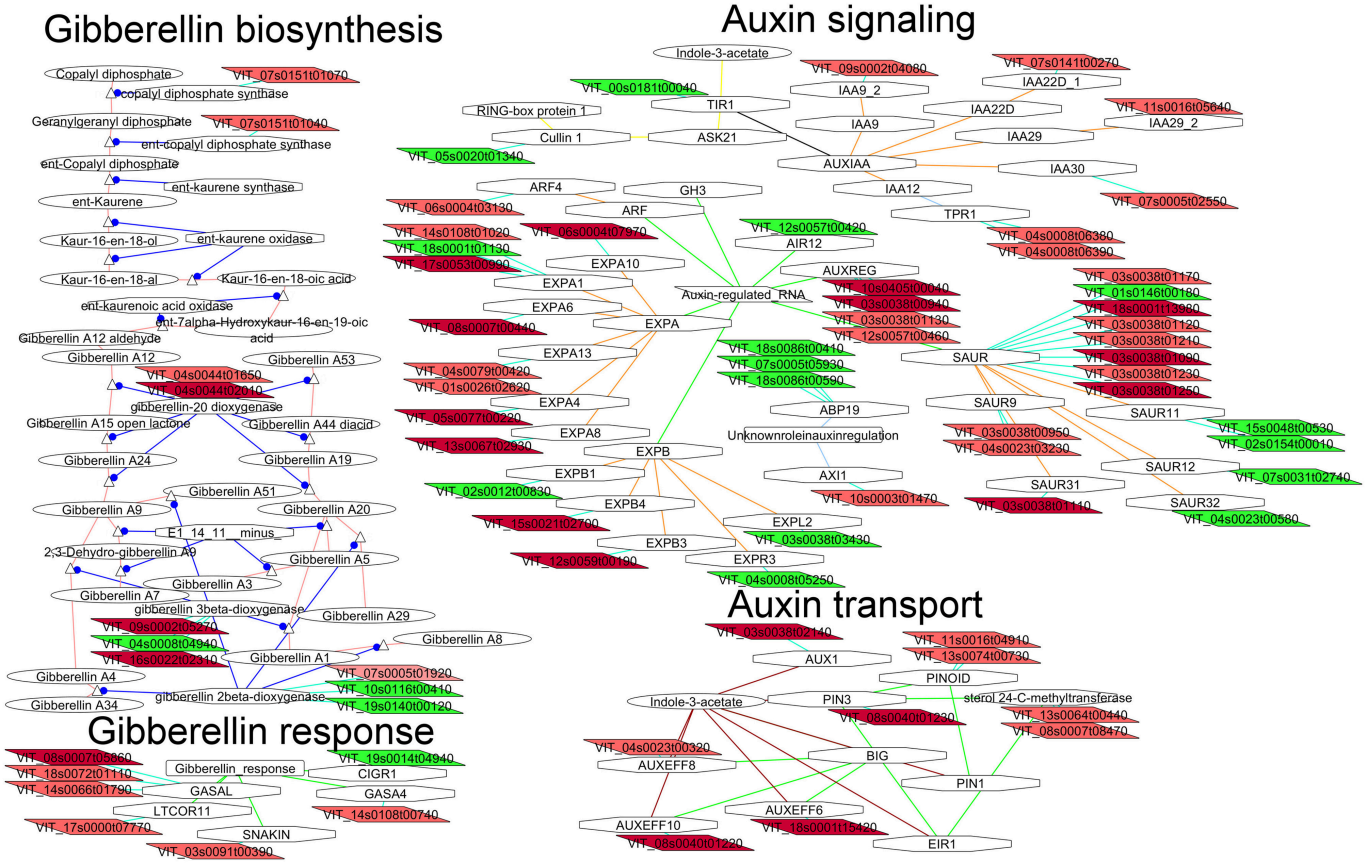
**Adapted Cytoscape networks including transcripts differentially expressed in flowers between loose and compact clones related to hormone biosynthesis, signaling, and transport**. Genes over-expressed in compact clones in all comparisons are in dark red. Genes over-expressed in compact clones in 2 or 3 comparisons are in red. Genes over-expressed in loose clones in all comparisons are in dark green. Genes over-expressed in loose clones in 2 or 3 comparisons are in green. Figure is adapted from networks 10904, 30010, 30003, and 50004 from Grimplet et al. (2009).

Networks were manually inspected to find those that presented relevant changes. These analyses allowed us to identify key networks and possible causes for cluster compactness as well as important information on early fruit development that will be discussed along this section (Figures 3–**6**, Supplementary Images 1–10). We observed changes in gene expression between compact and loose clones in flowers, and the clone with more and bigger berries (clone 1134) showed more differences with the loose clones than the other compact clone. It was however difficult to clearly distinguish if differences in cell replication or timing impacted fruit set (and thus berry number), the number of cells (berry size), or both. We identified four main categories of genes showing differential expression, related to: cellular activity, pathogens interaction, hormonal response and phenylpropanoids biosynthesis.

#### Loose and compact clones show great difference in flower transcriptome indicating a distinct cell division rate and/or asynchronous development

Comparison between flowers of clones producing tight clusters and clones producing loose clusters indicated a distinct cell division rate and/or asynchronous development. Most noticeably genes related to a greater activity in production of cellular material were more abundant in the compact clones. Evidences were specifically gathered at the level of carbohydrate and nucleic acid metabolism as well as the regulation of cell cycle and cell division.

##### Carbohydrate metabolism. cell wall

The composition and size of the fruits as they grow are very dependent of the efficiency of the flower as a nutrient sink (Bihmidine et al., 2013) and significant differences were observed between compact and loose clones in the carbohydrate metabolism in flower.

Important regulators of the sucrose metabolism (Figure 3) were seen to have isogenes specifically expressed in flower. Most noticeably, cell wall invertase (VIT_04s0008g01140) had greater expression in the loose clones than in clone 1134 and a vacuolar form was more expressed in the compact clones than in clone 368 (VIT_16s0022g00670). The cell-wall forms have been associated with rapidly growing tissues (Eschrich, 1980), they were induced by wounding and pathogenic attack (Sturm and Chrispeels, 1990), and have been implicated in phloem unloading and source/sink regulation (Eschrich, 1980; Roitsch et al., 1995). Gene expression in flower also indicated that starch seems to be preferentially catabolized into dextrin and maltodextrin with the increase of expression of several isogenes of alpha-(7 isoforms) and beta-amylases (2 isoforms) in the loose clones with respect to clone 1134. Higher expression of starch synthase (VIT_00s1488g00020) might indicate greater starch production in loose clones. Additionally a possible regulator of amylases (Liu and Thornburg, 2012), a transcript homologous to Myb305, was more abundant in the loose clone 368 vs. compact clones (VIT_14s0083g01060, Figure 3). However, the change of carbohydrate and cell osmolarity might be reminiscent of the flower opening mechanism (van Doorn and Van Meeteren, 2003) thus it would maintain turgor in the flowers of the loose clone, indicating a slight difference in the timing (delay) in loose against compact clones. As mentioned above, this difference could not be phenotyped since the samples were in an equivalent external stage: flowers were sampled at the end of flowering, with fallen stamen.

The next step was to identify the potential fate of the carbohydrates that would be produced from the DEG in the compact clones. In plants, most of the carbon fixed by photosynthesis is incorporated into cell wall carbohydrates. Compact clones showed an increase of expression of several transcripts involved in the biosynthesis of compounds that might be related to an increase of cell wall material. Starting from the fructose, all the enzymes that are involved in the biosynthetic pathway of both D-mannose and GDP mannose (Figure 3) presented at least an isoform over-expressed in flowers of the compact clones. These included fructokinases (VIT_18s0089g01230, VIT_05s0102g00710), hexokinases (VIT_18s0001g14230), mannose-6-phosphate_isomerase (VIT_01s0011g03750), phosphomannomutase (VIT_01s0011g03750, only G1). Other genes that might be involved in the biosynthesis of the predominant cell wall components arabinose or UDP-xylose (Seifert, 2004) (Figure 3), such as UDP-glucuronate 4-epimerase (VIT_15s0048g00330, only G1), UDP-glucuronate decarboxylase (VIT_05s0077g02300) and alpha-N-arabinofuranosidase (VIT_08s0032g00890, VIT_12s0055g01180) were also more abundant in compact clones. In cell wall (Supplementary Image 1) many differences between the expression levels of isogenes were observed. There were a few families that seem to be specific to one or the other cluster type. One of them, the pectinacetylesterase, which is involved in the regulation of pectin acetylation, had three isogenes over-expressed in flowers of the compact clones vs. clone 368, as well as four isogenes of the fasciclin-like arabinogalactan proteins involved in cell adhesion (Johnson et al., 2003). They might also be involved in cell expansion, since a mutant was observed causing swelling in roots (Shi et al., 2003). To complete the picture related to cell wall, several major families of cell wall related proteins showed differential expression between isogenes in a large amount but were evenly represented between the loose and compact clones, amongst them, the pectin methylesterase inhibitors, the pectinesterases, the pectate lyases or the xyloglucan endotransglycosylases. These centrally important aspects of expansion are also mediated by auxin, which is critical for skin strength in the earliest stages in flowers (Reeves et al., 2012). Overall while the gene expression is contrasted between clones, no routes leading to specific cell wall metabolites emerged as specific in any cluster type probably because substrate specificity of isogenes is not yet well-described.

In summary, there are differences between loose and compact clones in the expression of genes related to carbohydrate and cell wall metabolism. It can be hypothesized that cells in the compact clones were dividing more actively, triggering a large cascade of events that would explain the high number of differentially expressed genes but will likely complicate the identification of the primary genetic factors initiating the events.

##### Purine and pyrimidine biosynthesis

Transcripts involved in the metabolism of nucleic acid components were another indicator of differences in cellular activity between compact and loose clones. Several genes related to purine metabolism (Figure 4) tended to be up-regulated in flowers of the compact clones. Genes coding for enzymes involved in the next part of the pathway were more clearly over-expressed in the flowers of the compact clones indicating greater production of deoxynucleotides. The ribonucleoside-diphosphate reductase presented three isogenes (VIT_07s0031g01990, VIT_14s0068g02000, VIT_07s0031g02000) over-expressed in the compact clones. The nucleoside-triphosphatases (VIT_19s0015g01800, VIT_10s0003g01720 over-expressed in compact clone 1134) were involved in the conversion of ATP and GTP into ADP and GDP for RNA biosynthesis. Adenine phosphoribosyltransferase (VIT_00s1847g00010), which is involved in the purine salvaging, was also over-expressed in flowers of the compact clones.

As observed for the purine metabolism pathway, an increase of the gene expression in compact clones was observed in the pyrimidine pathway (Figure 4) with few changes affecting the earlier biosynthesis steps. The changes specifically affected transcripts for the interconversion of nucleotides triphosphate toward the production of deoxynucleotide diphosphates. Activity related to DNA repair was observed by the increase of dUDP pyrophosphatase (Dubois et al., 2011) and then production of dUMP. These observations might be linked to processes in the replication and repair related networks. In addition, transcripts of genes coding for enzymes involved in the pyrimidine salvaging pathways were also more abundant in compact clones, such as cytidine deaminase (VIT_17s0000g01370) and thymidine kinase (VIT_12s0057g00500).

The VitisNet analysis indicates significant differences in the nucleotide metabolism pathway between flowers of compact and loose clones. It suggests that these differences were not affecting the *de novo* biosynthesis of the nucleotides but may be related to interconversion and salvaging. These results shall be put in perspective with observations in the networks related to genetic regulation, more specifically DNA repair.

##### Regulation of DNA replication and repair mechanisms

The rate of DNA repair is dependent on many factors, including cell type, cell age and extracellular environment. In the studied clones at E-L 26 we observed a greater activity of this pathway in compact clones. Most of the DEG involved in DNA replication (twenty; Figure 5) were more abundant in the compact clones, like other pathways related to replication and repair. One of the six identified MCM genes related to DNA replication has previously been shown to be up-regulated after fertilization (Dresselhaus et al., 2006). MCM6 is essential for both vegetative and reproductive growth and development in plants (Dresselhaus et al., 2006).

Base excision repair (Figure 5) is the predominant DNA damage repair pathway for the processing of small base lesions. A large portion of the genes (10 over 30 genes) belonging to this network was more abundant in the compact clones. The differentially expressed transcripts were found either in the network common branches or in the mechanism of reparation of segments of 2–13 nucleotides.

DNA mismatch repair (Figure 5) is a highly conserved biological pathway that plays a key role in maintaining genomic stability (Li, 2008). Several of the over-expressed genes in the compact clones also belonged to the DNA polymerase complex. Homologous recombination (Figure 5) is essential for the accurate repair of DNA double-strand breaks (potentially lethal lesions), and acts before the cell enters mitosis. Once again, over-expression of genes related to DNA replication and repair in flowers of compact clones is another marker of a more active cell division compared to loose clones.

##### Cell cycle and regulation of actin cytoskeleton

Transcripts of about a third of the genes (97/322) involved in the cell cycle network (Supplementary Image 2) were more abundant in compact clones. The observation of a greater expression of these genes, combined with transcript abundance related to DNA processing mechanisms was another indicator of a more active division state in the compact clones. In addition, the expression of the genes involved in the network of regulation of actin cytoskeleton (Supplementary Image 3) was more abundant in flowers of the compact clones (82 of 343 genes).

Overall, differences were observed between compact and loose clones of Garnacha Tinta in the expression of cell division-related genes (carbohydrate and nucleic acid metabolism as well as regulation of cell cycle and cell division). This could be due to a difference in the rate of fecundated flowers in the two clone types, resulting in the comparison of a pool with a greater ratio of fecundated flowers (in the compact clones) vs. a pool with a lower ratio (in the loose ones) that would be in different cell division states. At this point of the development, cell division is in an exponential phase (Harris et al., 1968; Ojeda et al., 1999) and slight differences could significantly be reflected in the transcriptome. This would eventually lead to the greater number of berries observed in the compact clones. In addition, the differential activity in terms of cell replication could lead to a differential final cell number in the berries, and ultimately to a different berry size. In comparison G1-26, this could explain the differences in berry size between clone 1134 and clone 368 (Supplementary Table 1). Since few differences were observed in networks related to cell expansion (the other cell growth mechanism in berries), it would be the differential number of cells what affect final berry size in this case.

The differences observed in the cell division-related gene expression could also be due to a slight delay in the development progress in the loose clone with respect to the compact one. Given that the berries derived from flowers that opened first (“first berries”) have less probability to abscise than the later opening flowers (Kühn et al., 2014), a delay in the development could produce a greater berry abscission rate, thus affecting berry number. Unfortunately it was not possible to determine if the transcriptome differences were due to only one or the two possible causes proposed.

#### Plant pathogen interaction and relation to the jasmonate/methyl jasmonate interconversion

DEG involved in the mRNA surveillance pathway were predominantly expressed in the loose clones. They belonged more specifically to pre-mRNA 3′-end processing machinery and non-sense-mediated decay (NMD). Some of them, such as SMG7 (VIT_00s0527g00010, VIT_00s0640g00020) appear to regulate the expression of the genes involved in pathogen response in *Arabidopsis* (Rayson et al., 2012). Therefore, the expression of genes related to the plant-pathogen interaction was further examined.

Salicylic acid (SA) is a signal molecule involved in interactions between plants and pathogens. Enzymes potentially involved in its biosynthesis pathway did not exhibit differential expression of their corresponding transcripts. However, some genes involved in SA signaling were differentially expressed in flowers and that are known to be involved in pathogen response. Homologous to EDS1 (4 adjacent isogenes on the genomic sequence) were over-expressed in flowers of the loose clones vs. clone 1134. These genes were known to be involved in R protein-mediated signaling (Dempsey et al., 2011). Twenty R proteins (Supplementary Image 4) presented more abundant transcripts in flowers of the loose clones. In the plant-pathogen interaction network (Supplementary Image 5), several isoforms of BAK1 (8 over 19) and EIX1/2 (7/20) genes were more abundant in flowers of the loose clones vs. clone 1134. These genes are known to act together in the plant defense against pathogens induced by ethylene (Bar et al., 2010). Differences in expression of genes potentially regulated by ethylene (Supplementary Image 6) were observed in both compact and loose clones but members of Ethylene Response Factor subfamily were clearly more abundant in the loose clones. These genes corresponded to the subfamily IX or B-3 according to Nakano et al. (2006). The genes in group IX have often been linked in defensive gene expression in response to pathogen infection (Berrocal-Lobo et al., 2002) and this group contains PTI genes (Gu et al., 2002) that were known to be regulated by EDS1 (Dempsey et al., 2011). The WRKY transcription factors can also play a role in the defense mechanism (Rushton et al., 2010) and many of them were over-expressed in loose clones (20 genes).

Transcript level-related evidence of differential accumulation of jasmonic acid was unclear since expression of different transcripts coding for proteins involved in its biosynthesis in the alpha-linolenic acid metabolic pathway was increased in compact or loose clones. The isoforms of jasmonate O methyltransferase/VIT_04s0023t03810 VIT_04s0023t03800 VIT_04s0023t03790) were over-expressed in the flowers of the compact clones and the methyl jasmonate esterases (VIT_00s0253t00170, VIT_00s0253t00160 VIT_00s0253t00150) were preferentially expressed in the loose clones. The first enzyme catalyzes the conversion of jasmonate to methyl-jasmonate (MEJA) and the esterase catalyzes the demethylation of methyl-jasmonate. Jasmonate needs to be in the demethylated form to trigger defense response to herbivores (Wu et al., 2008), while MEJA is most likely involved in plant morphology.

There is no obvious reason explaining the greater expression of genes potentially involved in pathogen interaction in loose clusters, but both (pathogen-related gene expression and cluster loosening) could be consequences of the flower abscission process. The activation of different defense responses at flower abscission zones was described in tomato (Meir et al., 2011). Grapevine inflorescences treated to increase flower abscission showed up-regulation of pathogenesis-related genes (Domingos et al., 2016).

#### Gibberellin and auxin biosynthesis and signaling were likely to play a role in compact clones

##### Gibberellins

Application of gibberellins (GA) on the clusters is widely used in the table grape industry to control fruit set, elongate rachis or increase berry size (Coombe, 1960). It has different effects depending on the treatment concentration and timing. When applied at bloom, gibberellins affect fruit set and berry size (Dokoozlian and Peacock, 2001). We hypothesize that differences in the gibberellins metabolism or signaling would be observed at flowering between compact and loose clones in flowers of clones differing in berry number (and berry size in G1). Several transcripts coding for enzymes involved in GA biosynthesis (diterpenoid biosynthesis, Figure 6) were more abundant in flowers of the compact clones in the comparison G1-26, such as copalyl diphosphate synthase (VIT_07s0151g01070 loose clones vs. 1134), ent-kaurene synthase (VIT_07s0151g01040 loose clones vs. 1134), gibberellin-20 oxidase (VIT_04s0044g01650 loose clones vs. 1134, VIT_04s0044g02010) and the regulator BME3 (VIT_13s0019g04390 only G1). Moreover, flowers of the loose clones showed higher expression of transcript coding for the enzyme converting active GAs (GA1, GA3, GA4, and GA7) to inactive GAs (GA34, GA8): GA2-oxidase (VIT_10s0116g00410, VIT_19s0140g00120 loose clones vs. 1134). These findings are in agreement with Giacomelli et al. (2013) proposing that the pool of bioactive GAs in grapevine flowers during flowering and fruit set is controlled by a fine regulation of the abundance and localization of GA oxidase transcripts.

Genes involved in GA signaling (Figure 6) did not show differential expression between compact and loose clones in flowers but several genes known to be regulated by GA showed greater expression in the compact clones. Interestingly, several GASA-like transcripts showed preferential expression in the compact clones (VIT_08s0007g05860, VIT_18s0072g01110, VIT_14s0066g01790, VIT_03s0091g00390, and VIT_14s0108g00740). GASA proteins are involved in diverse processes, and GASA4 in *Arabidopsis* is present in flower and involved in the seed development and yield (Roxrud et al., 2007). One of the transcripts (VIT_03s0091g00390) corresponds to the SNAKIN subfamily which is known to be an antimicrobial (Segura et al., 1999) but more recently its role in the cell division was described (Nahirñak et al., 2012).

##### Auxin

In grapevine, auxin is a growth factor required for fruit growth. No significant observation could be made on auxin biosynthesis related transcripts to identify a possible greater production in compact or loose clones. More significantly, transcripts involved in the auxin transport (Figure 6) were more abundant in flowers of the compact clones, such as PINOID (VIT_11s0016g04910, VIT_13s0074g00730) and the auxin efflux carriers PIN3 (VIT_08s0040g01230, VIT_17s0000g02420) PIN6 (VIT_18s0001g15420) PIN5 (VIT_04s0023g00320) PIN10 (VIT_08s0040g01220), and AUX1 (VIT_03s0038g02140). As mentioned above, it has been recently shown that berries derived from flowers that open first have less probability to abscise than the flowers that open later, and that this ability requires decreased ethylene-related gene expression dependent on polar auxin transport (Kühn et al., 2014). Later, Kühn et al. (2016) found that polar auxin transport and transcripts of four putative PIN genes decreased in conjunction with increased abscission, and the inhibition of polar auxin transport resulted in fruit drop. In this context, over-expression of auxin transporter genes could be related to a greater final number of berries in the cluster by contributing to lower the number of abscised flowers or fruitlets.

In the auxin regulation pathway (Figure 6), transcripts coding for proteins related to the early response to auxin were up-regulated in flowers of the compact clones, including six transcripts for AUX/IAA and seven transcripts for SAUR. Quantitatively, ARF6 was one of the most differentially expressed genes in the G4 comparison (G4-03 and G4-26). ARF6 is known to be present in the flower and embryo, and in *Arabidopsis* it was specifically localized in the lower tier of the embryo and suspensors (Rademacher et al., 2011). Recently, Su et al. (2016) found that ARF6 and ARF8 are required in Arabidopsis for gradient auxin response and can mediate auxin-induced gene activation in somatic embryogenesis induction. In tomato, down-regulation of ARF6 and ARF8 by microRNA 167 led to floral development defects and female sterility (Liu et al., 2014). ARF4 was the second Auxin Response Factor over-expressed in the compact clone 1134 (in G1-26). It has been characterized in tomato fruit (Sagar et al., 2013), where lowers chloroplast production and starch and is down-regulated by presence of sugars. The expression of ARF4 in tomato increases between anthesis and 4 days post-anthesis and might be involved in fruit set (Zouine et al., 2014). In grapevine ARF4 is more abundant in high seed content berries at ripening (Gouthu and Deluc, 2015).

Cross-talk between GAs and auxins has proven to play an important role during fruit set in tomato via the activation of GA biosynthtetic enzyme GA20 oxydase by auxin (de Jong et al., 2009) two transcripts coding for GA20ox are over-expressed in compact clones (Figure 6). In grapevine crosstalk beween these two hormones is also critical in flower set initiation and parthenocarpy (Jung et al., 2014; Lu et al., 2016).

#### Genes involved in phenylpropanoids biosynthesis show that important secondary metabolites might be specifically expressed within clones

A significant number of genes involved in the biosynthesis of phenylpropanoids, flavonoids and anthocyanins were differentially expressed between cluster types, although most of them showed isogenes preferentially expressed in either compact or loose clones. There were also differences in the transcript abundance of genes affecting the production of several important secondary metabolites. All but three of the 46 stilbene synthase genes were preferentially expressed in flowers of the loose clones vs. clone 1134 (Supplementary Image 7). It was shown that over-expression of grapevine stilbene synthase VIT_16s0100g00910 can induce parthenocarpy in tomato (Ingrosso et al., 2011) and thus this gene might be related to the control of berry number. These authors also observed that greater amounts of stilbene were related to pollen sterility.

Several transcripts coding for enzymes potentially involved in the anthocyanin biosynthesis showed preferential expression in flowers of compact clones (Supplementary Image 9), including three Anthocyanidine rhamnosyl-transferase (VIT_00s0820g00020, VIT_15s0046g01950, VIT_00s0218g00140) and three Anthocyanidin 3-O-glucoside-6”-O-malonyltransferase (VIT_12s0134g00660, VIT_12s0134g00620, VIT_12s0134g00640). The latter two were up-regulated in clusters with small berries. The earlier steps in the phenylalanine biosynthesis (Supplementary Image 10) also showed a greater gene expression in the flowers of the compact clones, including shikimate dehydrogenase (VIT_14s0030g00650, VIT_14s0030g00660), shikimate kinase (VIT_18s0001g01730), and prephenate dehydratase (VIT_10s0116g01670).

## Conclusions

The characterization of the differential expression in clones of Garnacha Tinta presenting phenotypic differences in traits related to cluster compactness allowed us to identify networks and candidate genes potentially involved in those traits. The flowers at the end of bloom seem to be an organ and developmental stage of crucial importance for the traits studied, while much less differences were observed in spring buds and young berries. Our study focused on the end of flowering which is a particularly active period of rapid changes but other stages could also play important role in compactness and a fine monitoring of the flowering stages would improve our knowledge. In the case of the analysis on berry, the microclimate caused by different compactness levels may also influence the genes expression and make more difficult the discrimination between genetics and environmental factors. All the stable differential traits considered (berry number, seed number and berry size), are potentially affected by the magnitude of cell division rate, and many related gene networks showed different expression levels, indicating a greater division rate in compact clones with more berries (and eventually more seeds or larger berries). Differential expression of transcripts involved in hormone signaling and transport support that auxin and gibberellins play a central role in fruit set, and some identified key genes have been noted. Other hormones, such as ethylene and jasmonate may differentially regulate potential indirect effects, such as the activation of some defense mechanism or polyphenols production.

## Author contributions

JG performed the gene expression analysis and interpretation. JT and NL performed phenotyping analysis. JI designed the study. JG and JI drafted the manuscript. All authors read and approved the final manuscript.

## Funding

This work was financially supported by the projects AGL2014-59171R (co-funded by FEDER) and AGL2010-15694 and the Ramon y Cajal grant RYC-2011-07791, all from the Spanish MINECO. JT was the recipient of a predoctoral fellowship from MINECO (Grant: BES-2011-047041).

### Conflict of interest statement

The authors declare that the research was conducted in the absence of any commercial or financial relationships that could be construed as a potential conflict of interest. The reviewer SDS and handling Editor declared their shared affiliation, and the handling Editor states that the process nevertheless met the standards of a fair and objective review.

## Supplementary material

Supplementary Image 1 **Adapted Cytoscape networks including transcript differentially expressed in flowers between loose and compact clones related to cell wall metabolism**. Genes over-expressed in compact clones in all comparisons are in dark red. Genes over-expressed in compact clones in 2 or 3 comparisons are in red. Genes over-expressed in loose clones in all comparisons are in dark green. Genes over-expressed in loose clones in 2 or 3 comparisons are in green. Figure is adapted from networks 40006 from Grimplet et al. (2009).

Supplementary Image 2 **Adapted Cytoscape networks including transcript differentially expressed in flowers between loose and compact clones related to cell cycle**. Genes over-expressed in compact clones in all comparisons are in dark red. Genes over-expressed in compact clones in 2 or 3 comparisons are in red. Genes over-expressed in loose clones in all comparisons are in dark green. Genes over-expressed in loose clones in 2 or 3 comparisons are in green. Figure is adapted from networks 44110 from Grimplet et al. (2009).

Supplementary Image 3 **Adapted Cytoscape networks including transcript differentially expressed in flowers between loose and compact clones related to regulation of actin cytoskeleton**. Genes over-expressed in compact clones in all comparisons are in dark red. Genes over-expressed in compact clones in 2 or 3 comparisons are in red. Genes over-expressed in loose clones in all comparisons are in dark green. Genes over-expressed in loose clones in 2 or 3 comparisons are in green. Figure is adapted from networks 44810 from Grimplet et al. (2009).

Supplementary Image 4 **Adapted Cytoscape networks including transcript differentially expressed in flowers between loose and compact clones related to R proteins from plant–pathogen interaction**. Genes over-expressed in compact clones in all comparisons are in dark red. Genes over-expressed in compact clones in 2 or 3 comparisons are in red. Genes over-expressed in loose clones in all comparisons are in dark green. Genes over-expressed in loose clones in 2 or 3 comparisons are in green. Figure is adapted from networks 34627 from Grimplet et al. (2009).

Supplementary Image 5 **Adapted Cytoscape networks including transcript differentially expressed in flowers between loose and compact clones related to plant–pathogen interaction**. Genes over-expressed in compact clones in all comparisons are in dark red. Genes over-expressed in compact clones in 2 or 3 comparisons are in red. Genes over-expressed in loose clones in all comparisons are in dark green. Genes over-expressed in loose clones in 2 or 3 comparisons are in green. Figure is adapted from networks 34626 from Grimplet et al. (2009).

Supplementary Image 6 **Adapted Cytoscape networks including transcript differentially expressed in flowers between loose and compact clones related to ethylene signaling**. Genes over-expressed in compact clones in all comparisons are in dark red. Genes over-expressed in compact clones in 2 or 3 comparisons are in red. Genes over-expressed in loose clones in all comparisons are in dark green. Genes over-expressed in loose clones in 2 or 3 comparisons are in green. Figure is adapted from networks 30008 from Grimplet et al. (2009).

Supplementary Image 7 **Adapted Cytoscape networks including transcript differentially expressed in flowers between loose and compact clones related to phenylpropanoids biosynthesis**. Genes over-expressed in compact clones in all comparisons are in dark red. Genes over-expressed in compact clones in 2 or 3 comparisons are in red. Genes over-expressed in loose clones in all comparisons are in dark green. Genes over-expressed in loose clones in 2 or 3 comparisons are in green. Figure is adapted from networks 10940 from Grimplet et al. (2009).

Supplementary Image 8 **Adapted Cytoscape networks including transcript differentially expressed in flowers between loose and compact clones related to flavonoids biosynthesis**. Genes over-expressed in compact clones in all comparisons are in dark red. Genes over-expressed in compact clones in 2 or 3 comparisons are in red. Genes over-expressed in loose clones in all comparisons are in dark green. Genes over-expressed in loose clones in 2 or 3 comparisons are in green. Figure is adapted from networks 10941 from Grimplet et al. (2009).

Supplementary Image 9 **Adapted Cytoscape networks including transcript differentially expressed in flowers between loose and compact clones related to anthocyanin biosynthesis**. Genes over-expressed in compact clones in all comparisons are in dark red. Genes over-expressed in compact clones in 2 or 3 comparisons are in red. Genes over-expressed in loose clones in all comparisons are in dark green. Genes over-expressed in loose clones in 2 or 3 comparisons are in green. Figure is adapted from networks 10942 from Grimplet et al. (2009).

Supplementary Image 10 **Adapted Cytoscape networks including transcript differentially expressed in flowers between loose and compact clones related to ethylene signaling**. Genes over-expressed in compact clones in all comparisons are in dark red. Genes over-expressed in compact clones in 2 or 3 comparisons are in red. Genes over-expressed in loose clones in all comparisons are in dark green. Genes over-expressed in loose clones in 2 or 3 comparisons are in green. Figure is adapted from networks 10400 from Grimplet et al. (2009).

Supplementary Table 1 **Phenotypic data and pair-wise ***t***-tests of the selected clones during three seasons**.

Supplementary Table 2 **Gene expression values data**. Sheet 1: normalized data for each samples, value are expressed as log2 of intensity. Sheet 2: functional annotation. Sheet 3: significance of the differential expression, ratio of transcripts expression in each comparison, genes meeting the cutoff of fold change >2 and *P* < 0.05 are reported as 1 for compact clones and −1 for loose clones.
