# Supplementary figures and images for: Differences in Flower Transcriptome between Grapevine Clones Are Related to Their Cluster Compactness, Fruitfulness, and Berry Size
Source: Front Plant Sci. 2017 Apr 27;8:632. doi: 10.3389/fpls.2017.00632 (PMC5406470; doi:10.3389/fpls.2017.00632)

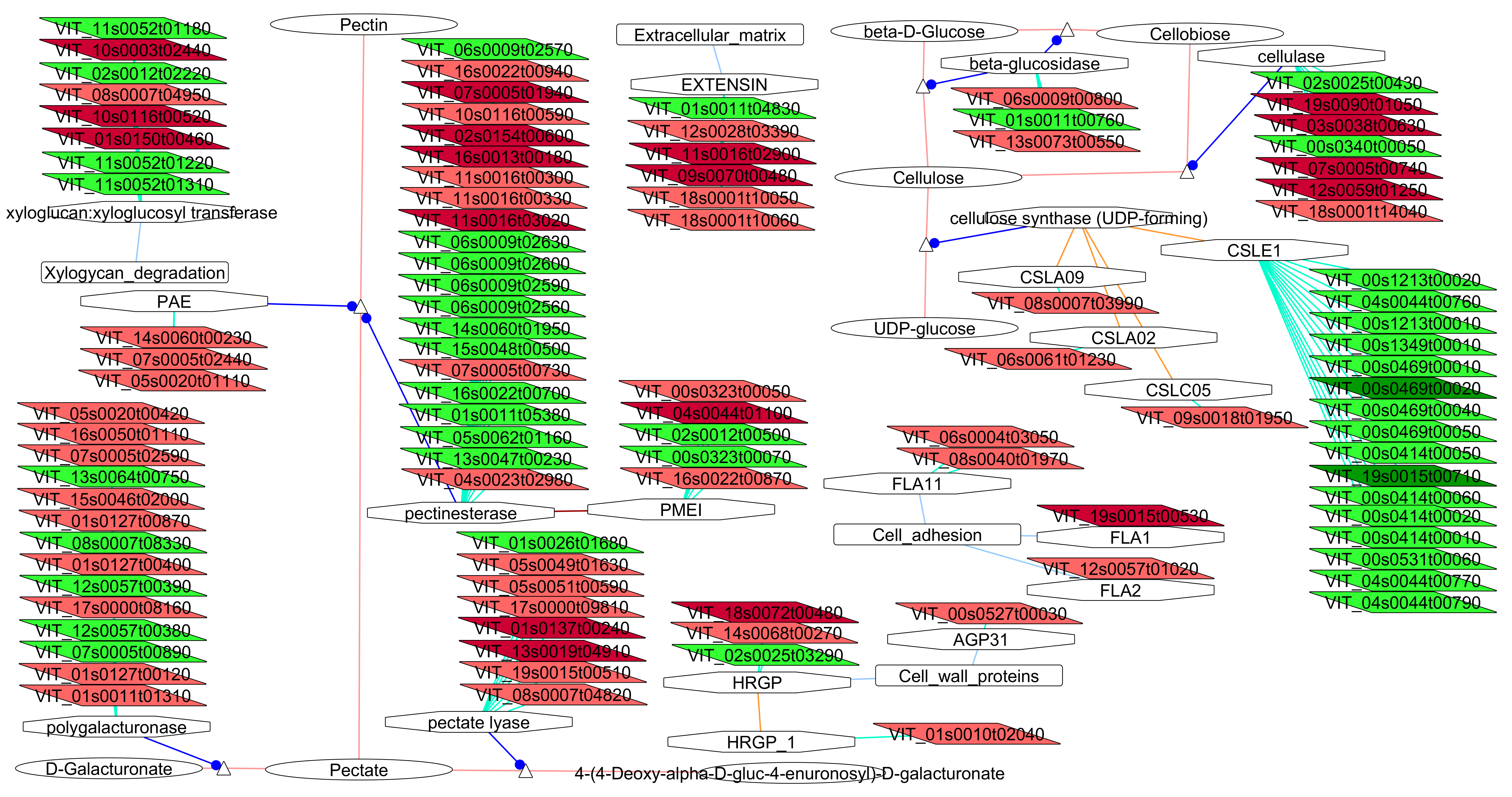

Supplement: Supplementary Image 1 — Adapted Cytoscape networks including transcript differentially expressed in flowers between loose and compact clones related to cell wall metabolism. Genes over-expressed in compact clones in all comparisons are in dark red. Genes over-expressed in compact clones in 2 or 3 comparisons are in red. Genes over-expressed in loose clones in all comparisons are in dark green. Genes over-expressed in loose clones in 2 or 3 comparisons are in green. Figure is adapted from networks 40006 from Grimplet et al. (2009). [file Image1.PNG]

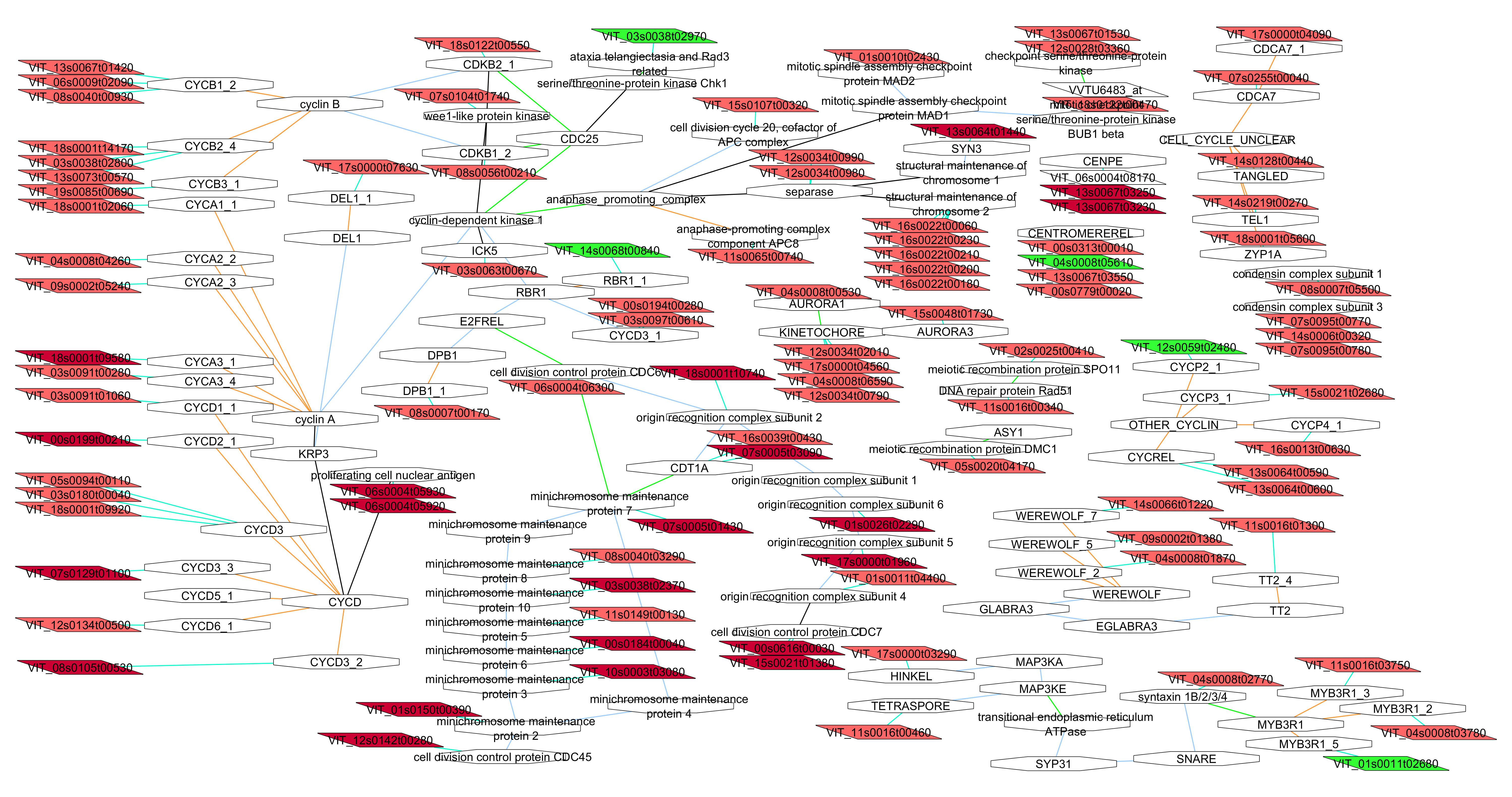

Supplement: Supplementary Image 2 — Adapted Cytoscape networks including transcript differentially expressed in flowers between loose and compact clones related to cell cycle. Genes over-expressed in compact clones in all comparisons are in dark red. Genes over-expressed in compact clones in 2 or 3 comparisons are in red. Genes over-expressed in loose clones in all comparisons are in dark green. Genes over-expressed in loose clones in 2 or 3 comparisons are in green. Figure is adapted from networks 44110 from Grimplet et al. (2009). [file Image2.PNG]

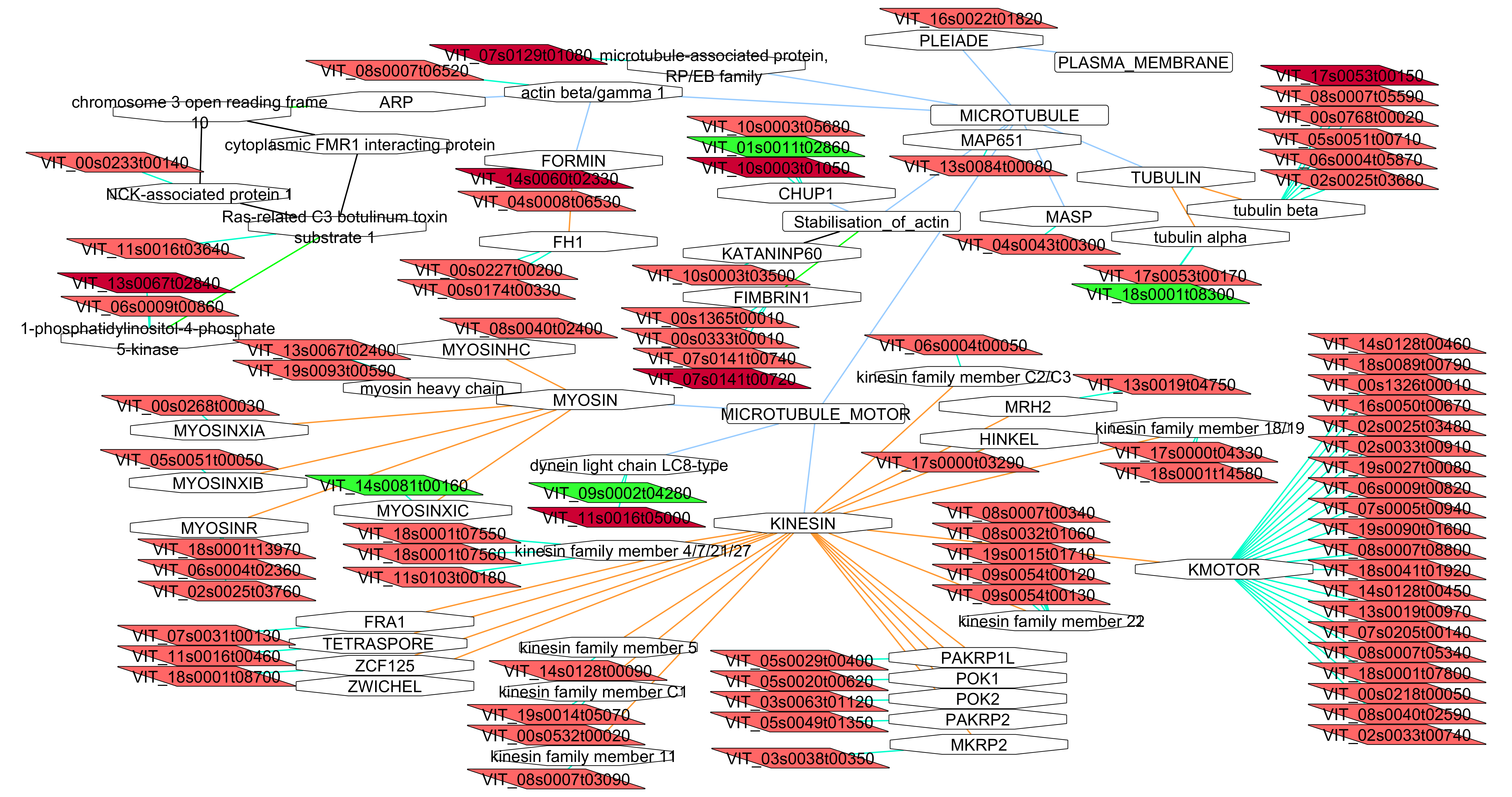

Supplement: Supplementary Image 3 — Adapted Cytoscape networks including transcript differentially expressed in flowers between loose and compact clones related to regulation of actin cytoskeleton. Genes over-expressed in compact clones in all comparisons are in dark red. Genes over-expressed in compact clones in 2 or 3 comparisons are in red. Genes over-expressed in loose clones in all comparisons are in dark green. Genes over-expressed in loose clones in 2 or 3 comparisons are in green. Figure is adapted from networks 44810 from Grimplet et al. (2009). [file Image3.PNG]

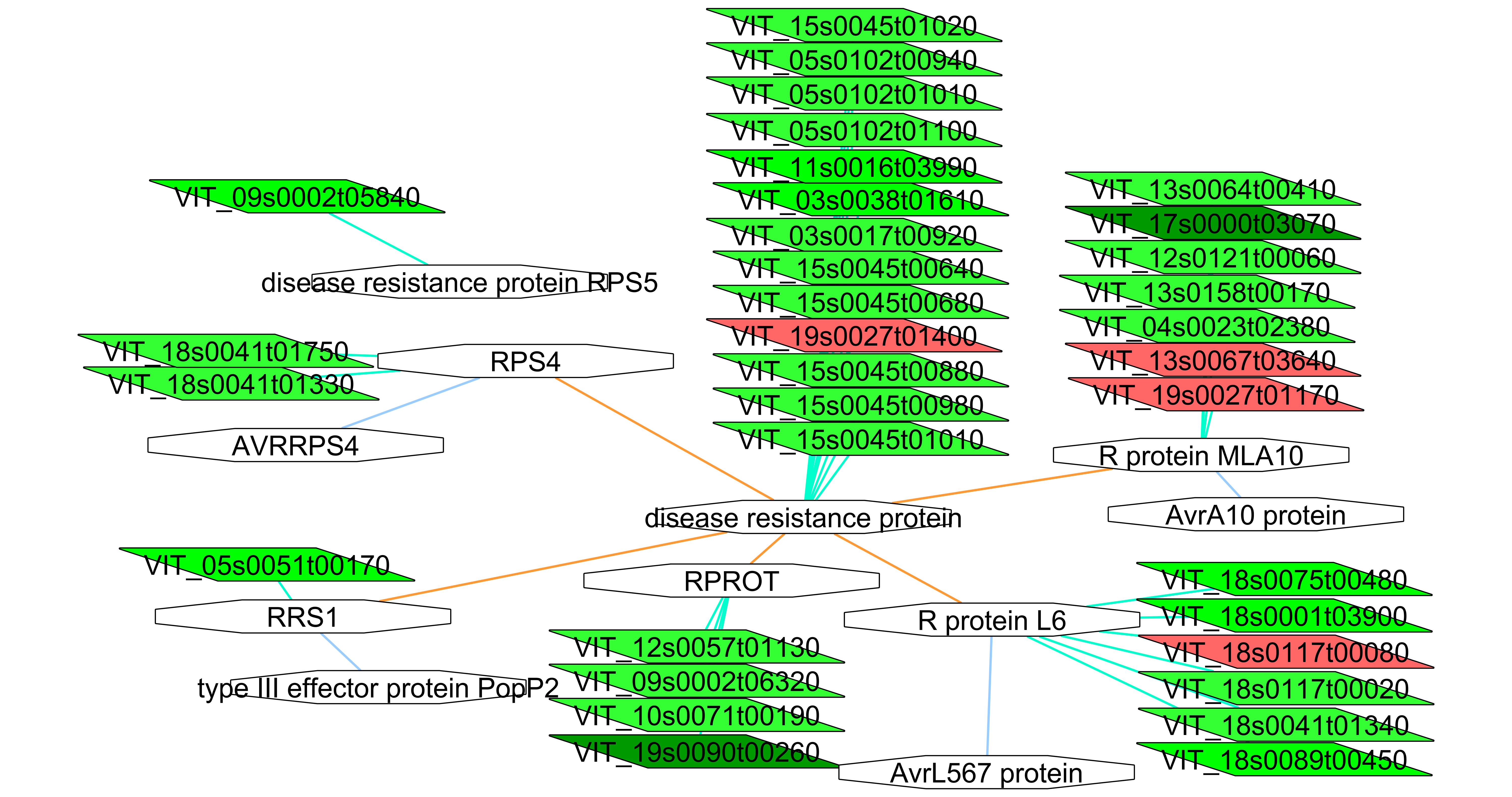

Supplement: Supplementary Image 4 — Adapted Cytoscape networks including transcript differentially expressed in flowers between loose and compact clones related to R proteins from plant–pathogen interaction. Genes over-expressed in compact clones in all comparisons are in dark red. Genes over-expressed in compact clones in 2 or 3 comparisons are in red. Genes over-expressed in loose clones in all comparisons are in dark green. Genes over-expressed in loose clones in 2 or 3 comparisons are in green. Figure is adapted from networks 34627 from Grimplet et al. (2009). [file Image4.PNG]

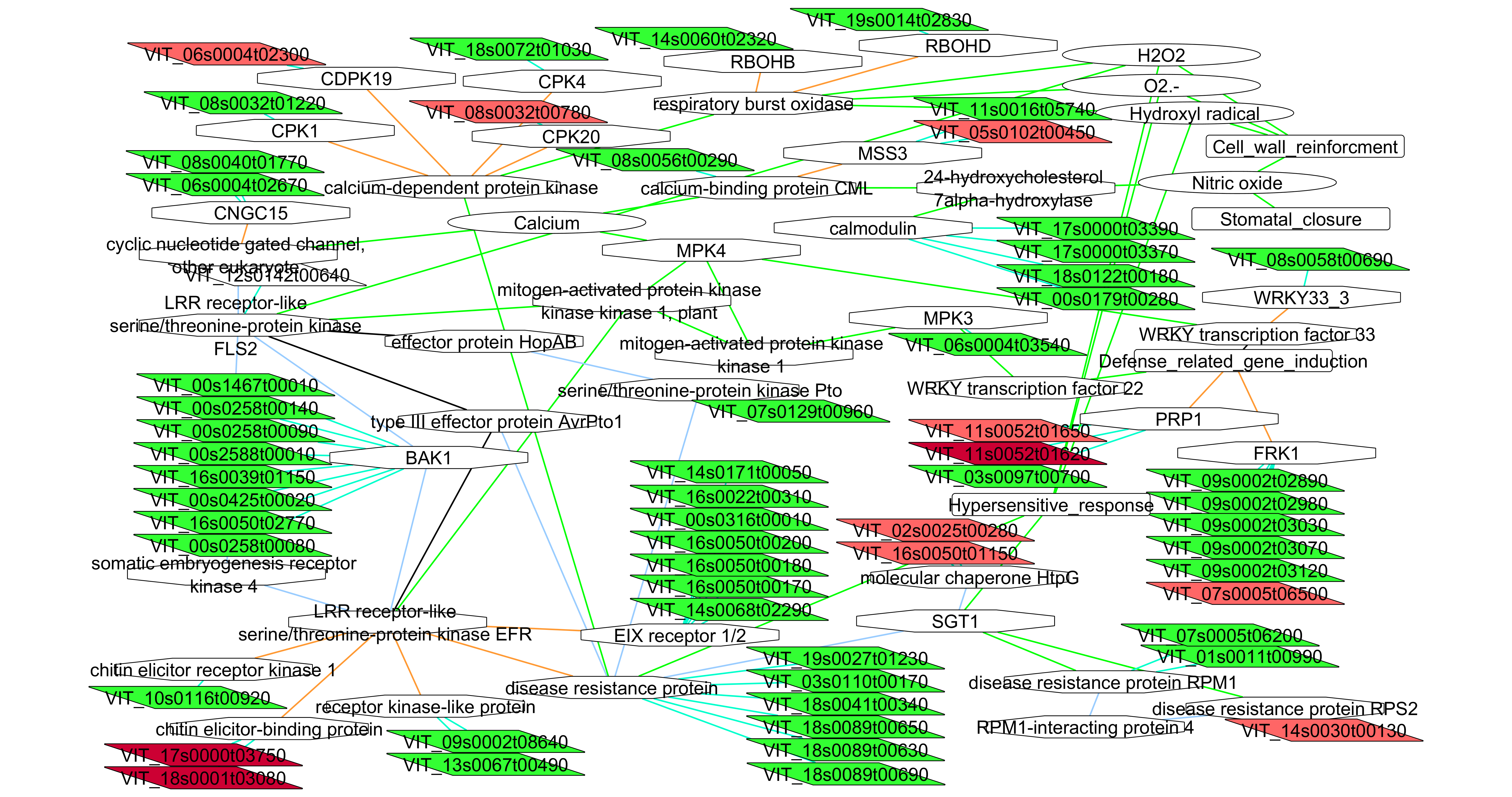

Supplement: Supplementary Image 5 — Adapted Cytoscape networks including transcript differentially expressed in flowers between loose and compact clones related to plant–pathogen interaction. Genes over-expressed in compact clones in all comparisons are in dark red. Genes over-expressed in compact clones in 2 or 3 comparisons are in red. Genes over-expressed in loose clones in all comparisons are in dark green. Genes over-expressed in loose clones in 2 or 3 comparisons are in green. Figure is adapted from networks 34626 from Grimplet et al. (2009). [file Image5.PNG]

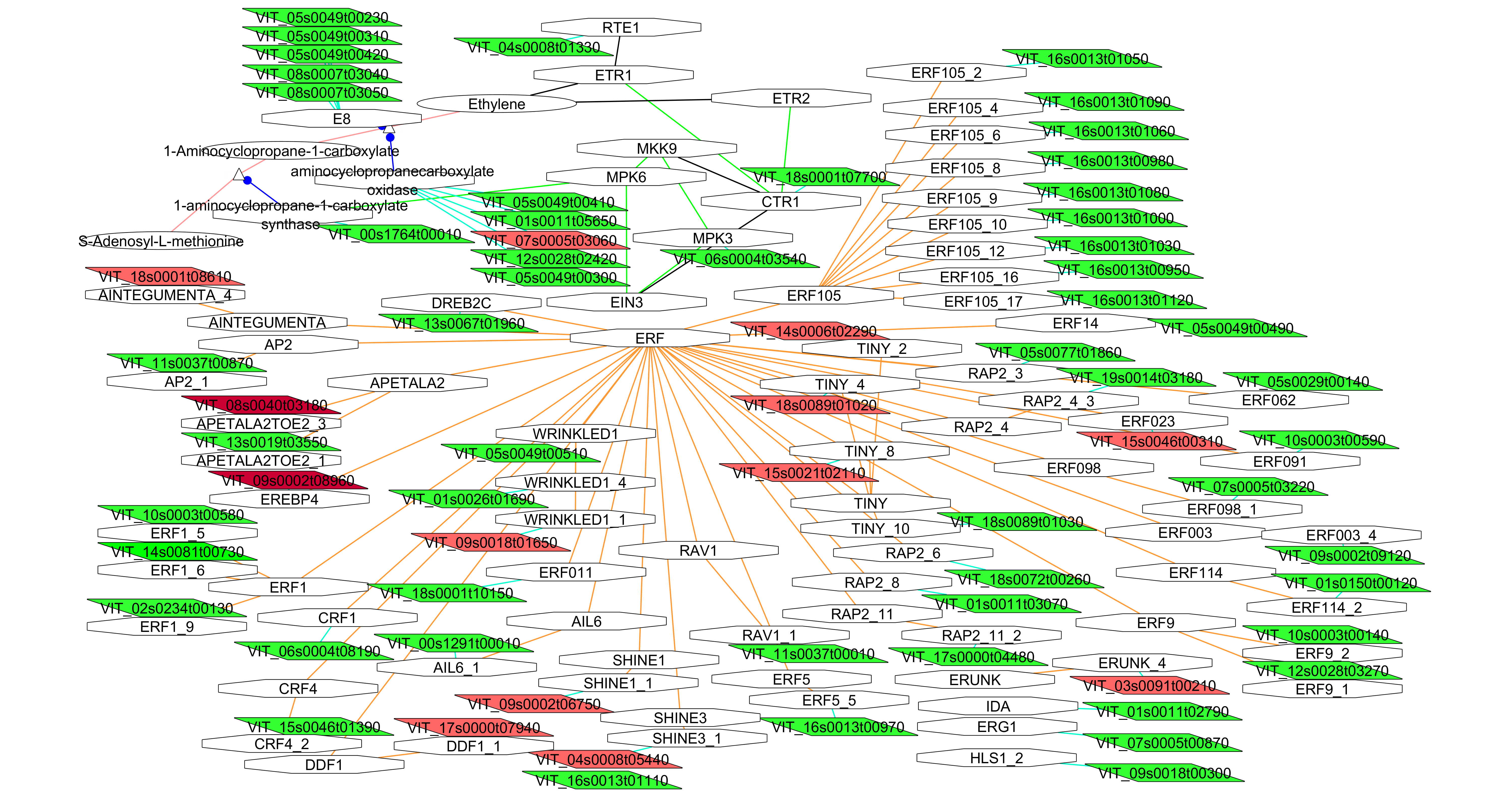

Supplement: Supplementary Image 6 — Adapted Cytoscape networks including transcript differentially expressed in flowers between loose and compact clones related to ethylene signaling. Genes over-expressed in compact clones in all comparisons are in dark red. Genes over-expressed in compact clones in 2 or 3 comparisons are in red. Genes over-expressed in loose clones in all comparisons are in dark green. Genes over-expressed in loose clones in 2 or 3 comparisons are in green. Figure is adapted from networks 30008 from Grimplet et al. (2009). [file Image6.PNG]

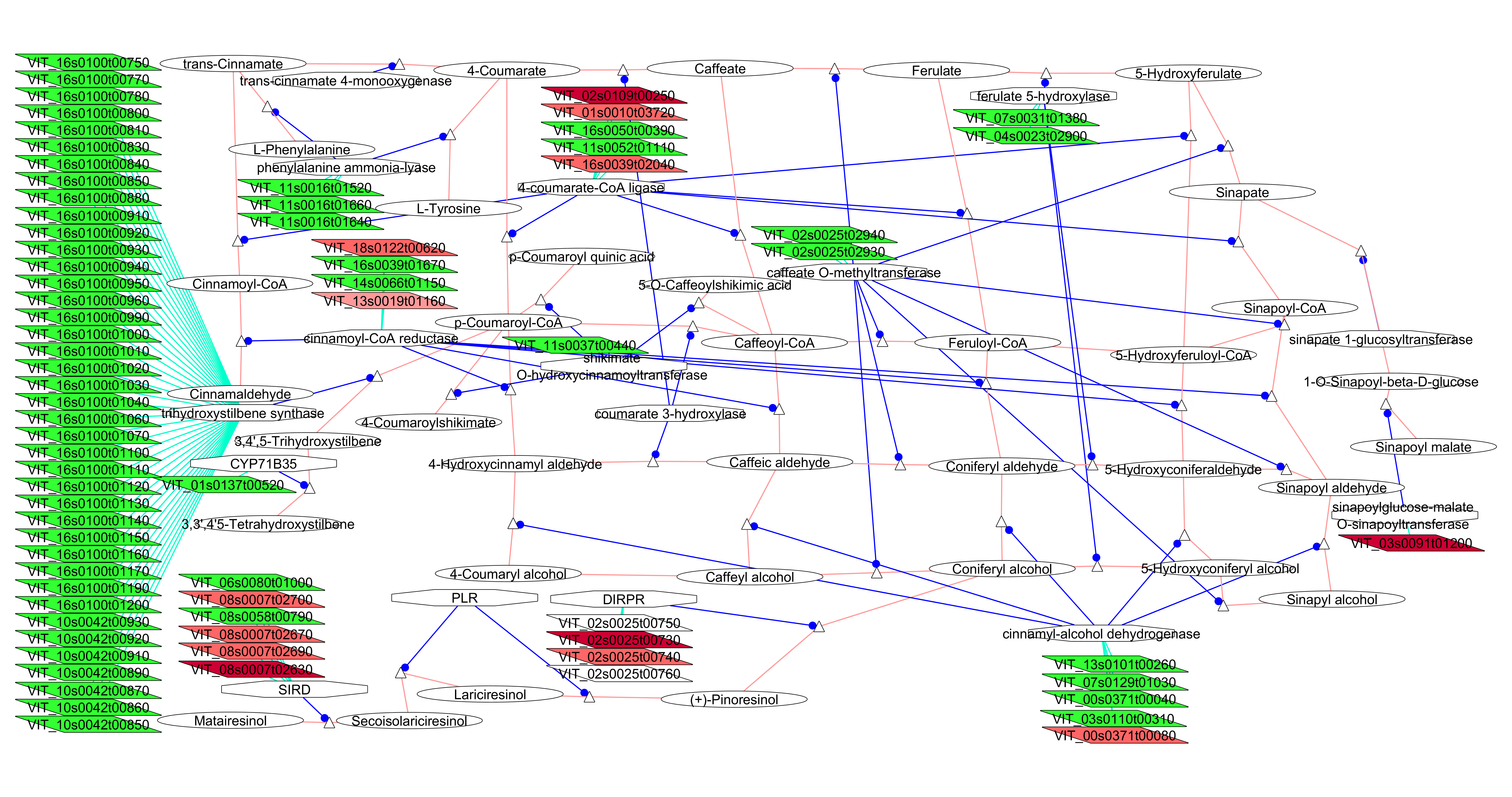

Supplement: Supplementary Image 7 — Adapted Cytoscape networks including transcript differentially expressed in flowers between loose and compact clones related to phenylpropanoids biosynthesis. Genes over-expressed in compact clones in all comparisons are in dark red. Genes over-expressed in compact clones in 2 or 3 comparisons are in red. Genes over-expressed in loose clones in all comparisons are in dark green. Genes over-expressed in loose clones in 2 or 3 comparisons are in green. Figure is adapted from networks 10940 from Grimplet et al. (2009). [file Image7.PNG]

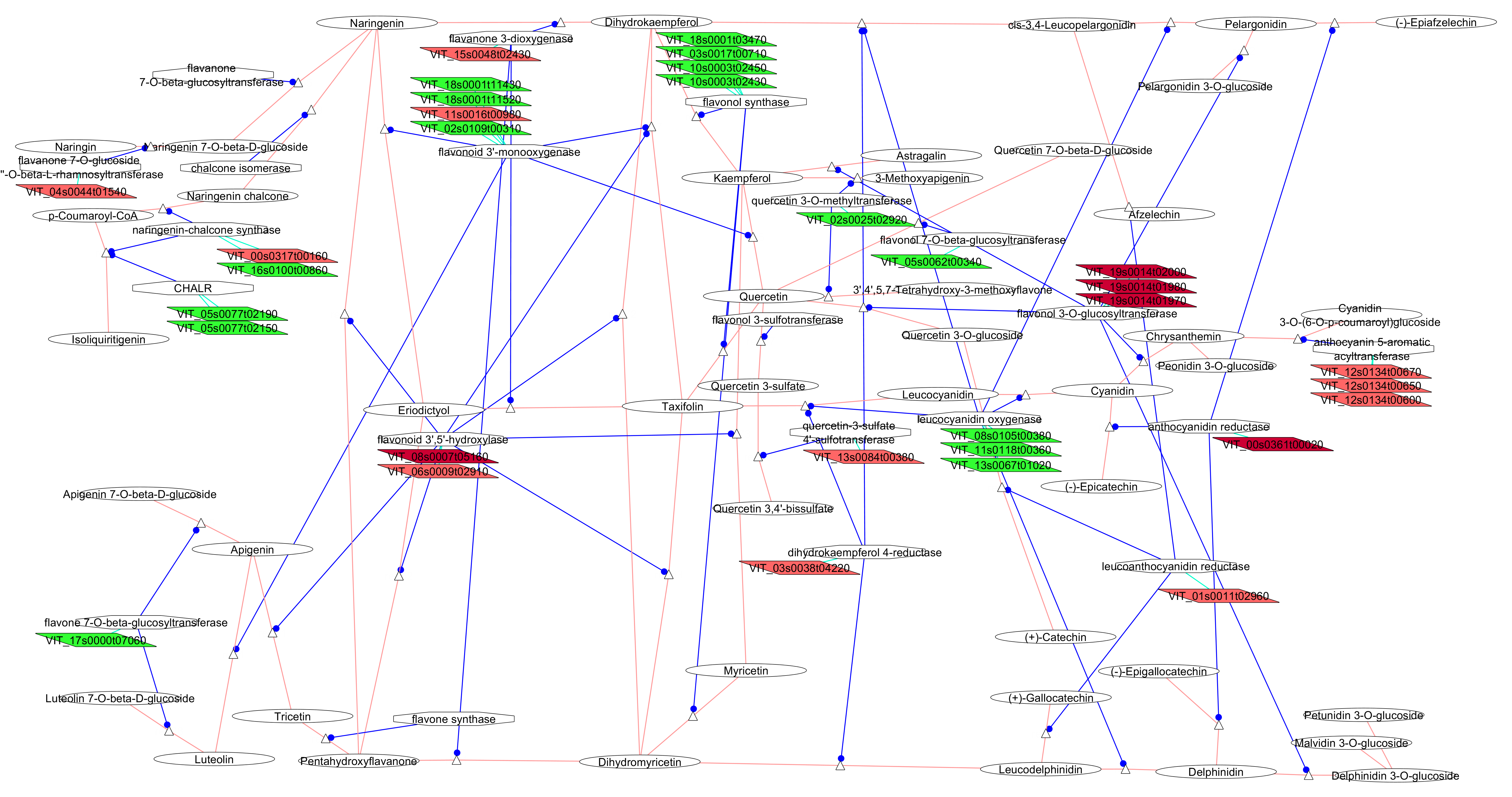

Supplement: Supplementary Image 8 — Adapted Cytoscape networks including transcript differentially expressed in flowers between loose and compact clones related to flavonoids biosynthesis. Genes over-expressed in compact clones in all comparisons are in dark red. Genes over-expressed in compact clones in 2 or 3 comparisons are in red. Genes over-expressed in loose clones in all comparisons are in dark green. Genes over-expressed in loose clones in 2 or 3 comparisons are in green. Figure is adapted from networks 10941 from Grimplet et al. (2009). [file Image8.PNG]

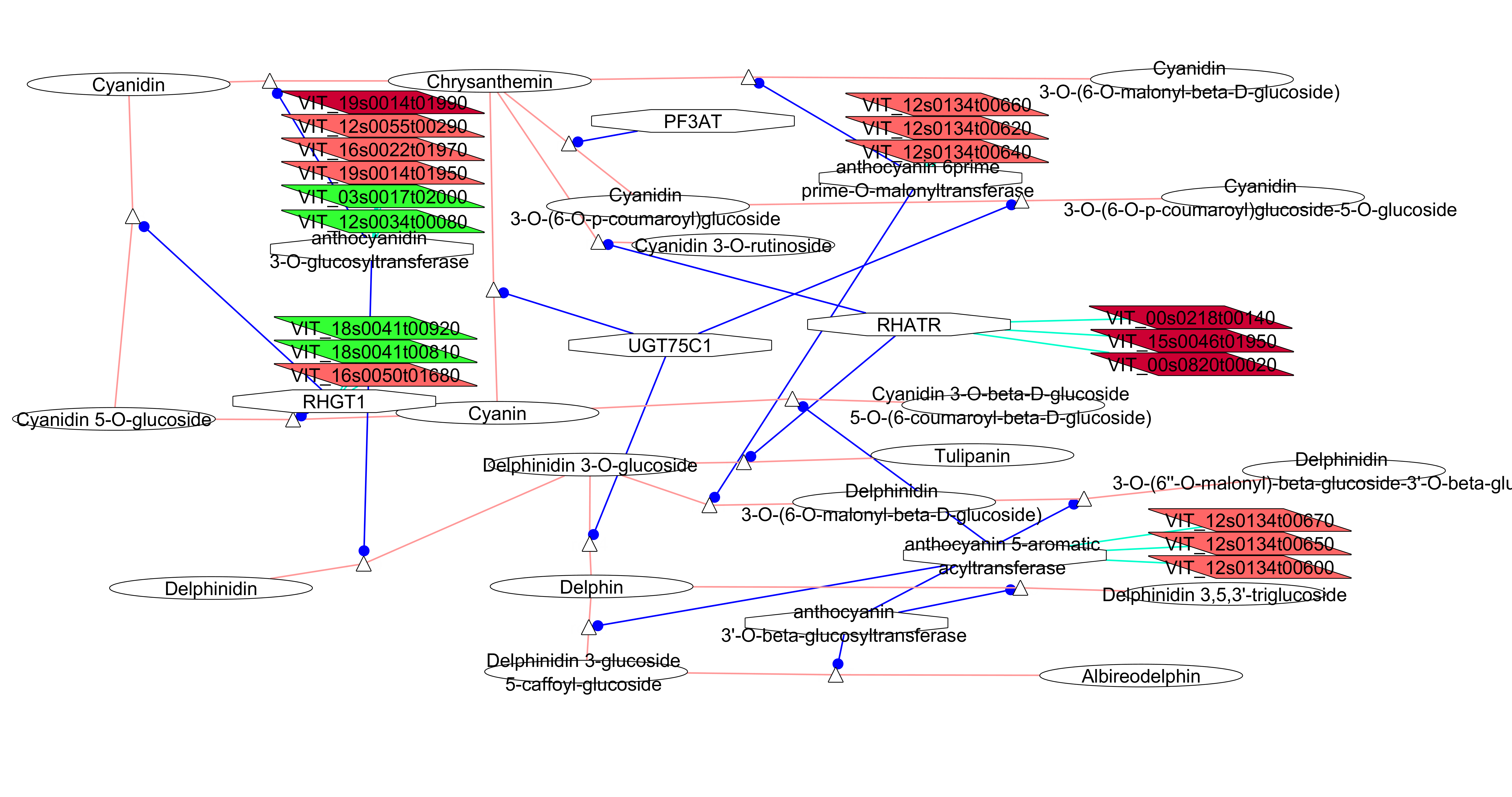

Supplement: Supplementary Image 9 — Adapted Cytoscape networks including transcript differentially expressed in flowers between loose and compact clones related to anthocyanin biosynthesis. Genes over-expressed in compact clones in all comparisons are in dark red. Genes over-expressed in compact clones in 2 or 3 comparisons are in red. Genes over-expressed in loose clones in all comparisons are in dark green. Genes over-expressed in loose clones in 2 or 3 comparisons are in green. Figure is adapted from networks 10942 from Grimplet et al. (2009). [file Image9.PNG]

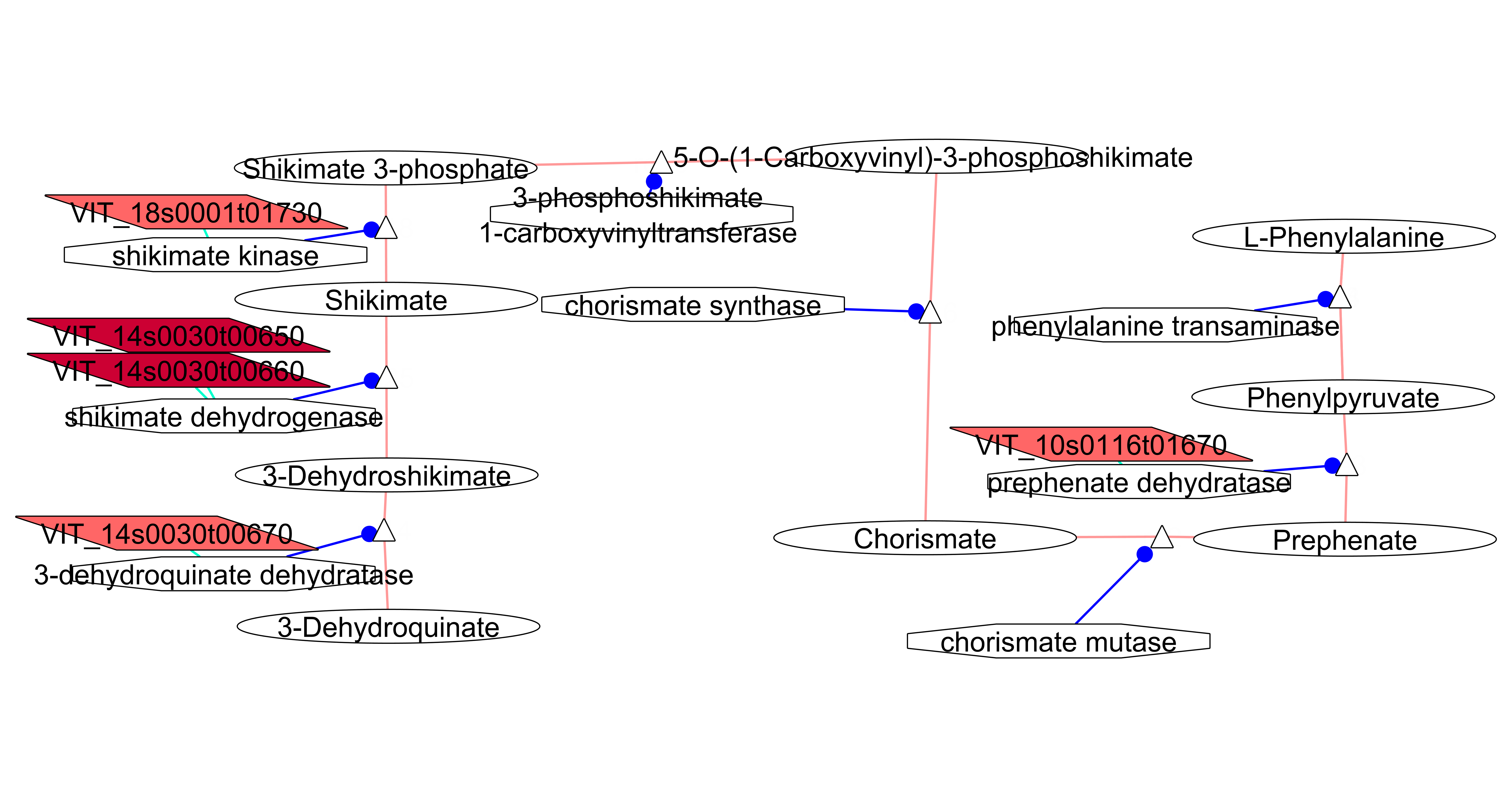

Supplement: Supplementary Image 10 — Adapted Cytoscape networks including transcript differentially expressed in flowers between loose and compact clones related to ethylene signaling. Genes over-expressed in compact clones in all comparisons are in dark red. Genes over-expressed in compact clones in 2 or 3 comparisons are in red. Genes over-expressed in loose clones in all comparisons are in dark green. Genes over-expressed in loose clones in 2 or 3 comparisons are in green. Figure is adapted from networks 10400 from Grimplet et al. (2009). [file Image10.PNG]
